# Physiological function of phospholipase D2 in anti-tumor immunity: regulation of CD8^+^ T lymphocyte proliferation

**DOI:** 10.1038/s41598-018-24512-x

**Published:** 2018-04-19

**Authors:** Van Ngo Thai Bich, Tsunaki Hongu, Yuki Miura, Naohiro Katagiri, Norihiko Ohbayashi, Yumi Yamashita-Kanemaru, Akira Shibuya, Yuji Funakoshi, Yasunori Kanaho

**Affiliations:** 1 Department of Physiological Chemistry, Faculty of Medicine and Graduate School of Comprehensive Human Sciences, University of Tsukuba, 1-1-1 Tennodai, Tsukuba, Ibaraki 305-8575 Japan; 2 Department of Immunology, Faculty of Medicine, University of Tsukuba, 1-1-1 Tennodai, Tsukuba, Ibaraki 305-8575 Japan; 3 Life Science Center for Survival Dynamics, Tsukuba Advanced Research Alliance (TARA), University of Tsukuba,, 1-1-1 Tennodai, Tsukuba, Ibaraki 305-8575 Japan

## Abstract

Two major phospholipase D (PLD) isozymes in mammals, PLD1 and PLD2, hydrolyze the membrane phospholipid phosphatidylcholine to choline and the lipid messenger phosphatidic acid. Although their roles in cancer cells have been well studied, their functions in tumor microenvironment have not yet been clarified. Here, we demonstrate that PLD2 in cytotoxic CD8^+^ T cells plays a crucial role in anti-tumor immunity by regulating their cell proliferation. We found that growth of tumors formed by subcutaneously transplanted cancer cells is enhanced in *Pld2*-knockout mice. Interestingly, this phenotype was found to be at least in part attributable to the ablation of *Pld2* from bone marrow cells. The number of CD8^+^ T cells, which induce cancer cell death, significantly decreased in the tumor produced in *Pld2*-knockout mice. In addition, CD3/CD28-stimulated proliferation of primary cultured splenic CD8^+^ T cells is markedly suppressed by *Pld2* ablation. Finally, CD3/CD28-dependent activation of Erk1/2 and Ras is inhibited in *Pld2-*deleted CD8^+^ T cells. Collectively, these results indicate that PLD2 in CD8^+^ T cells plays a key role in their proliferation through activation of the Ras/Erk signaling pathway, thereby regulating anti-tumor immunity.

## Introduction

The tumor microenvironment is comprised of cancer cells and numerous components such as blood vessels, fibroblasts, bone marrow-derived inflammatory cells, the extracellular matrix, and immune cells^1^. Under pathophysiological conditions, immune cells have reciprocal potentials being able to interfere with tumor development and promote the tumor growth. During tumor progression, effector cells of innate and adaptive immune responses recognize and attack tumor cells^2,3^, resulting in elimination of tumor cells from the human body. On the other hand, some other types of immune cells promote tumor growth by inhibiting anti-tumor immune responses, inducing inflammation and producing pro-tumoral cytokines and chemokines^3^. Lymphocytes infiltrate into tumor microenvironment of cancer patients and the presence of higher number of cytotoxic CD8^+^ T cells in solid tumors is associated with improved survival of patients with breast cancer, renal cell carcinoma, melanoma, and ovarian cancer^4,5^. Therefore, elucidation of the mechanisms for regulation of the development of T lymphocytes and their anti-tumor activity could provide insight into cancer immunotherapeutic opportunities.

Development of naïve T cells to effector T cells, which are capable of infiltrating into infected tissues or tumors to eliminate target cells, is initiated by engagement of the T cell receptor (TCR) of the naïve T cell and antigen-presenting cells: upon TCR activation in the lymphoid organs, naïve T cells, which are differentiated from precursor T cells in the thymus, undergo clonal expansion, proliferation and differentiation to become effector and memory T cells^6,7^. Engagement of TCR activates a number of downstream signaling pathways, such as MAPK^8^, NF-κB^6,9^, calcium^10^ and lipid signaling pathways, which control the developmental process from naïve T cells to effector T cells.

Two mammalian isozymes of phospholipase D (PLD), PLD1 and 2, which catalyze hydrolysis of membrane phosphatidylcholine (PC) to choline and the signaling lipid phosphatidic acid (PA), are involved in various cellular functions such as vesicular trafficking, the intracellular secretory response, cell motility and cell proliferation, through the product PA^11^. Several lines of evidence suggested that these PLDs are involved in immune responses and cancer progression. Primary macrophages lacking either PLD1 or PLD2 exhibit impaired phagocytosis and cell migration due to abnormal cytoskeletal organization^12^. PLDs are also suggested to function in Jurkat T cell activation^13^. Recently, we have reported that PLD1 in vascular endothelial cells is involved in tumor growth through the promotion of tumor angiogenesis in mice^14^. It has also been shown that expression of the inactive PLD2 mutant in EL4 lymphoma cells reduces their proliferation rate and invasiveness^15^. In addition, overexpression of PLD2 in low-invasive breast cancer cell line MCF-7 cells promotes tumor growth in severe combined immunodeficiency (SCID) mice, while silencing of PLD2 in highly-invasive breast cancer cell line MDA-MB-231 cells significantly abrogates lung metastases in SCID mice^16^, suggesting that PLD2 in these cancer cells plays a pivotal role in cancer progression. However, neither the involvement of PLD2 in the tumor microenvironment nor the link between PLD2-mediated immune responses and tumor immunity has been elucidated.

In the present study, we provide evidence that PLD2 positively regulates proliferation of CD8^+^ T cells, which have a critical function in cancer cell elimination, through TCR-dependent activation of the Ras-Erk signaling pathway, thereby contributing to the suppression of tumor progression.

## Result

### Ablation of *Pld2* promotes the tumor growth

**Figure 1:**
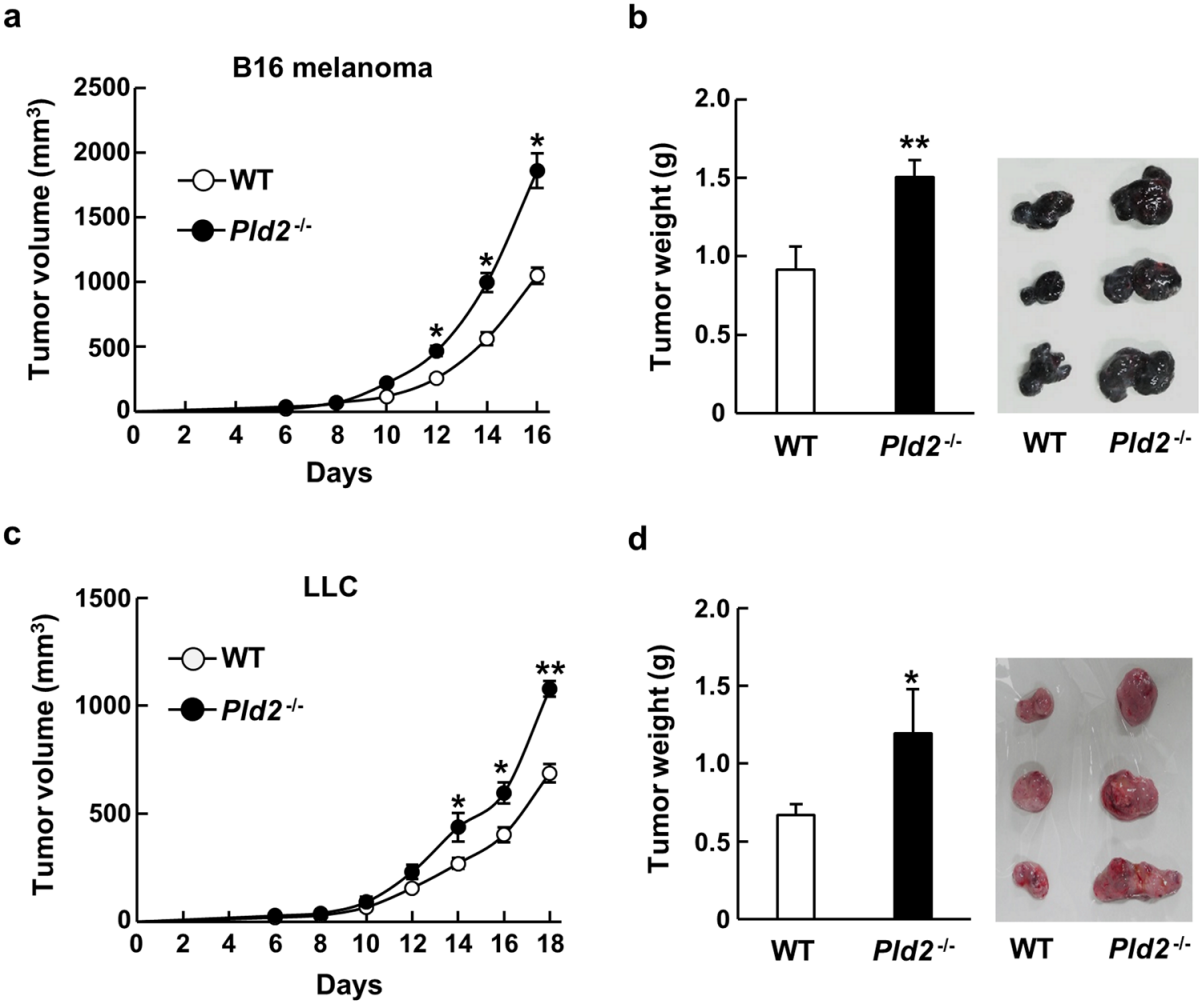
Tumor growth is enhanced in *Pld2*^−/−^ mice. B16 melanoma (B16) (**a**,**b**) and Lewis lung carcinoma (LLC) cells (**c**,**d**) were implanted into WT (n = 8) and *Pld2*^−/−^ mice (n = 6), and tumor volumes were measured every other day after sixth day of implantation (**a**,**c**). Tumors produced by B16 and LLC cells were dissected at 16 (**b**) and 18 days (**d**), respectively, and their weight was measured and shown as means ± SEM. *p < 0.05, **p < 0.01.

To explore the pathological roles of PLD2, we analyzed growth of tumors produced by implantation of B16 melanoma and Lewis lung carcinoma (LLC) cells into *Pld2*-knockout (*Pld2*^−/−^) mice, which had been generated in our laboratory^17^. Unlike the previous reports showing tumor-progressive functions of PLDs^14,16^, tumor growth was significantly enhanced in *Pld2*^−/−^ mice (Fig. 1a–d).

Since *Pld2*^−/−^ mice had been generated by replacing exons 14 and 15 of the *Pld2* gene^17^ (Supplementary Fig. S1), we cannot rule out the possibility that a partial peptide(s) of PLD2 is expressed in the mice and exerts an off-target effect. To exclude this possibility, we generated new *Pld2*-null mice (*Pld2*^−/−^*/*Cas9) with the CRISPR-Cas9 technique to delete most of the coding regions of PLD2 (Supplementary Fig. S2). Consistent with the result obtained with *Pld2*^−/−^ mice (Fig. 1), *Pld2*^−/−^*/*Cas9 mice implanted with B16 melanoma and LLC cells enhanced tumor growth (Supplementary Fig. S3), confirming that ablation of *Pld2* promotes the tumor growth.

### Ablation of *Pld2* inhibits tumor cell apoptosis without effect on tumor angiogenesis

**Figure 2:**
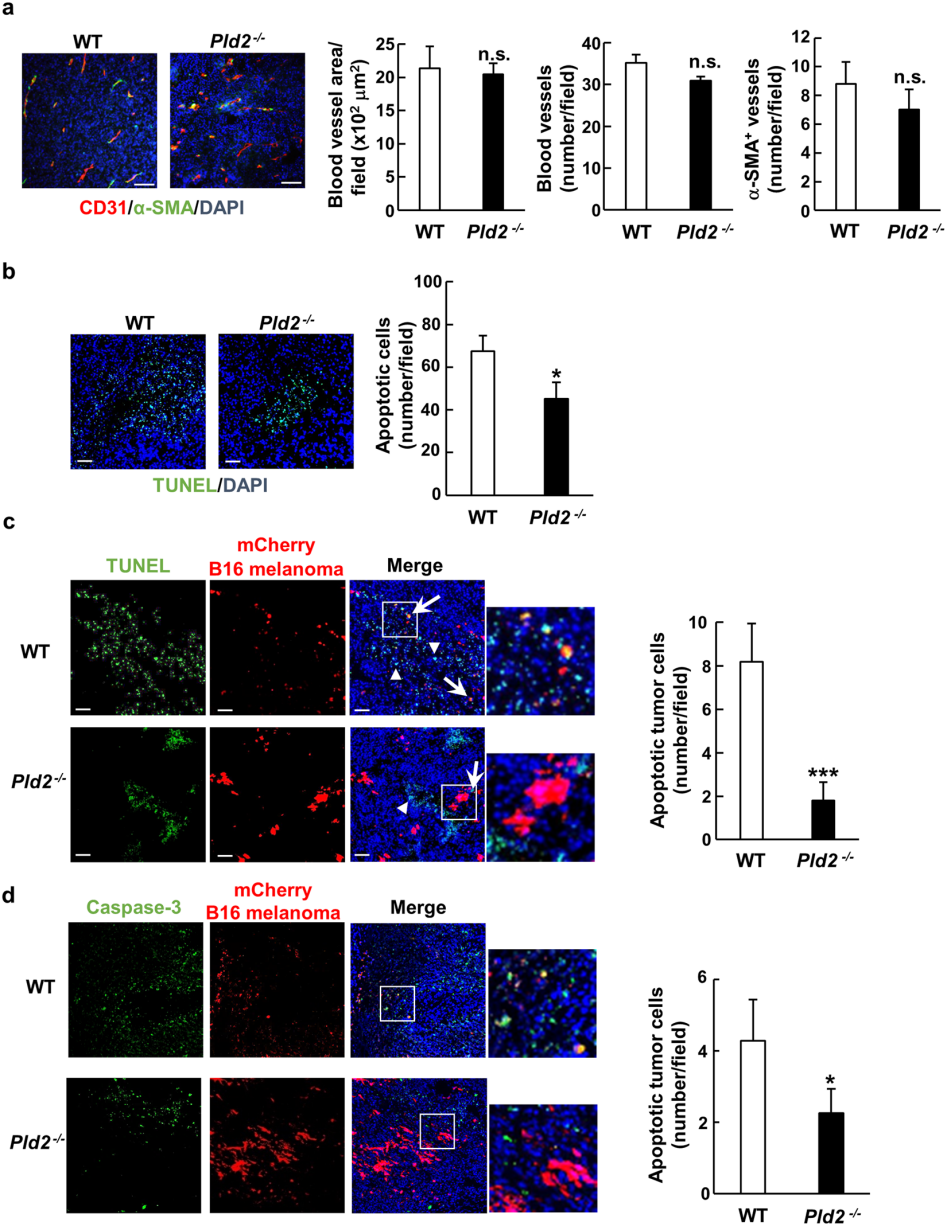
*Pld2* deletion suppresses apoptosis of tumor cells without effect on tumor angiogenesis. (**a**) Tumors produced by B16 melanoma cells were dissected at 16 days after implantation of the cell, and tumor sections were immunostained for CD31 (red) and alpha smooth muscle actin (α-SMA) (green) (left images). Nuclei were also stained with 4′,6-diamidino-2-phenylindole (DAPI) (blue). Blood vessel area (left graph) and number (middle graph), and number of mature vessels covered by α-SMA^+^ mural cells (right graph) were quantified (n = 6 for each genotype, at least 3 fields/section were captured). (**b**) Tumor sections were stained by the TUNEL method (green) and with DAPI (blue) (left images). Apoptotic cells in tumors were quantified (n ≥ 5 for each genotype). (**c**) B16 melanoma cells stably expressing mCherry (red) were implanted into WT and *Pld2*^−/−^ mice, and apoptotic cells in tumors were detected by TUNEL assay (left images, green) (n ≥ 5 for each genotype). Arrows represent apoptotic B16 melanoma cells and arrowheads apoptosis of mCherry-negative cells. Right panel shows the quantification of apoptotic B16 melanoma cells in the field. (**d**) Sections of tumors produced as in (**c**) were immunostained for cleaved caspase-3 (green), and nuclei were stained with DAPI (blue) (left images). Apoptotic B16 melanoma cells were quantified as in (**c**) (right panel). All quantification data represent means ± SEM. *p < 0.05; ***p < 0.0005; n.s., not significant. Scale bars, 50 μm.

Tumor angiogenesis is the critical event for tumor growth^18^. Although the previous studies have shown that PLD1 is involved in tumor angiogenesis^14^, ablation of *Pld2* did not affect tumor angiogenesis (Fig. 2a): immunofluorescence staining for the endothelial cell marker CD31 and the matured blood vessel marker α-smooth muscle actin (α-SMA) revealed that the number and area of blood vessels and number of matured vessels in B16 melanoma tumors formed in *Pld2*^−/−^ mice were comparable with those in wild-type (WT) mice. Thus, enhancement of tumor growth in *Pld2*^−/−^ mice is not related to tumor angiogenesis.

Imbalance between apoptosis and proliferation of tumor cells is a hallmark underlying tumor progression. TUNEL assay revealed less apoptotic cells in B16 melanoma tumors formed in *Pld2*^−/−^ mice (Fig. 2b). When B16 melanoma cells stably expressing the fluorescent protein mCherry were implanted, apoptosis of these cells in the tumor formed in *Pld2*^−/−^ mice was significantly suppressed (Fig. 2c), which was consistent with the result of immunostaining of cleaved caspase 3, a specific marker for apoptotic cells (Fig. 2d). These results indicate that death of tumor cells is inhibited in *Pld2*^−/−^ mice.

### PLD2 in CD8^+^ T cells is required for tumor growth suppression

**Figure 3:**
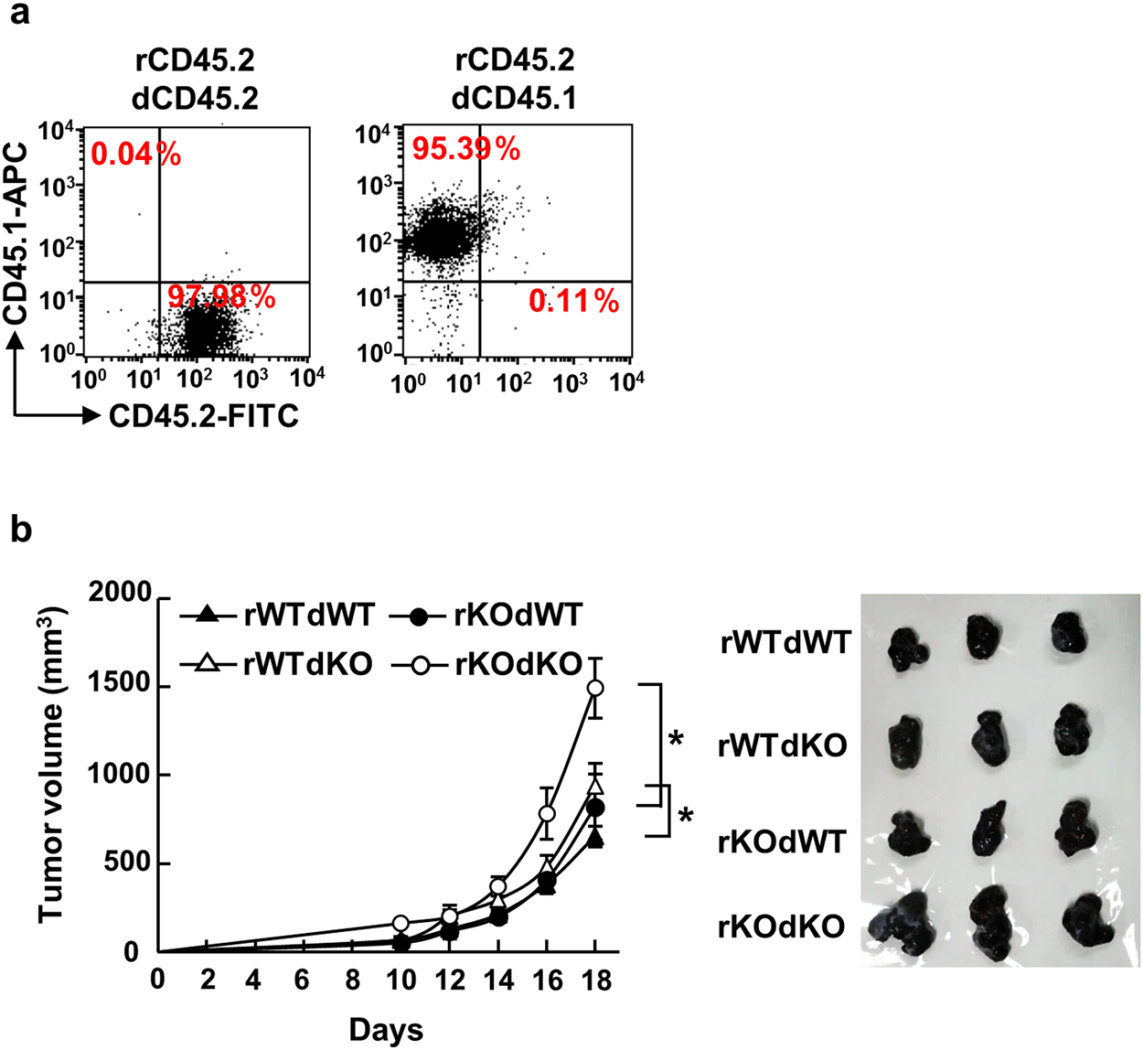
Deletion of *Pld2* from bone marrow cells promotes tumor growth. (**a**) Efficiency of bone marrow transplantation. Irradiated C57BL/6 mice with CD45.2-positive bone marrow cells were transplanted with CD45.2- (left) or CD45.1-positive bone marrow cells (right). Total bone marrow cells were isolated, and chimerism was analyzed by FACS using anti-CD45.1 and anti-CD45.2 antibodies. r, recipient; d, donor. (**b**) Tumor growth of B16 melanoma in WT and *Pld2*^−/−^ mice transplanted with WT and *Pld2*^−/−^ bone marrow cells. The pictures shown in the right are primary tumors dissected after 18 days of implantation. Data shown are means ± SEM (n = 6 for rWTdWT and rWTdKO; n = 5 for rKOdWT and rKOdKO). *p < 0.05.

For cancer cells to proliferate and form solid tumors, they have to overcome an anti-tumor immune response coordinated by innate and adaptive immune cells^19^. This concept and results shown above led us to assume that *Pld2*^−/−^ mice are defective for the anti-tumor immune system. To evaluate this assumption, we conducted bone marrow transplantation experiments. Bone marrow reconstitution efficiency was more than 95% as assessed by percentage of CD45.1-expressing donor haematopoietic stem cells in CD45.2^+^ recipient mice (Fig. 3a). Consistent with the result with *Pld2*^−/−^ mice (Fig. 1a,b), growth of the tumor produced by B16 melanoma cells in *Pld2*^−/−^ mice grafted with *Pld2*^−/−^ bone marrow cells (rKOdKO) was significantly enhanced compared with that in WT mice grafted with control bone marrow cells (rWTdWT) (Fig. 3b). Tumor growth in WT mice transplanted with *Pld2*^−/−^ bone marrow cells (rWTdKO) was promoted compared with that in rWTdWT. In addition, reconstitution of WT bone marrow cells into *Pld2*^−/−^ mice (rKOdWT) significantly suppressed the tumor-progressive phenotype of rKOdKO mice. These results demonstrate that PLD2 in bone marrow cells is crucial for tumor growth suppression. However, extent of tumor growth in rWTdKO was less than that in rKOdKO (Fig. 3b), indicating that PLD2 in other cell types distinct from bone marrow cells is also involved in the tumor growth suppression. This is supported by the results that *Pld2*^−/−^ mice reconstituted with WT bone marrow cells (rKOdWT) tended to slightly augment tumor growth compared with rWTdWT (Fig. 3b). Nevertheless, these results provide evidence that PLD2 in bone marrow-derived cells at least in part functions in anti-tumor immune responses.

**Figure 4:**
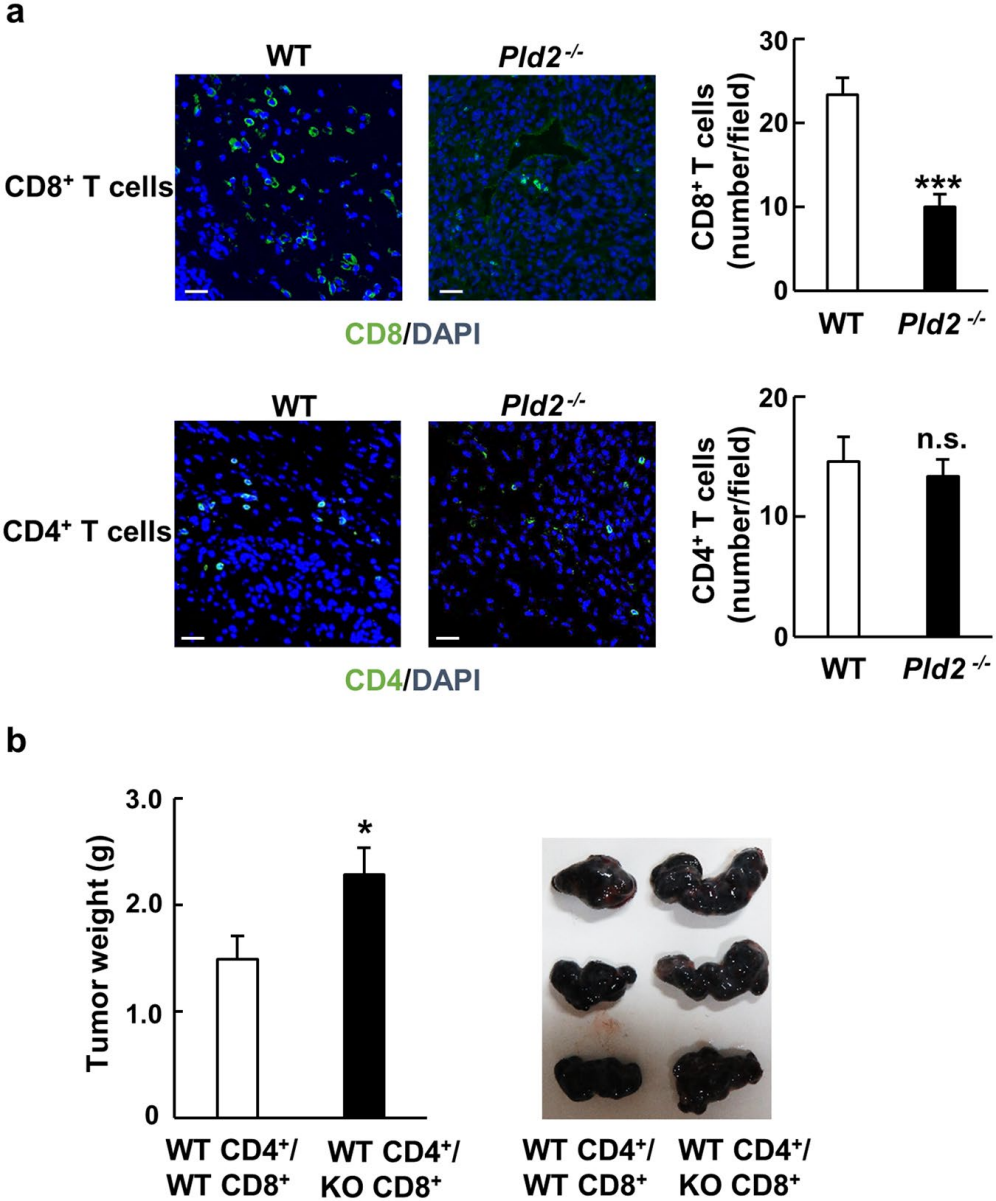
Ablation of *Pld2* in CD8^+^ T cells suppresses their infiltration into tumors and promotes tumor growth. (**a**) Infiltration of T cells into tumors formed in WT and *Pld2*^−/−^ mice was analyzed by immunostaining for CD8 (upper images) and CD4 (lower images). The number of T cells was counted (right panels) (n = 6 for each phenotype, at least 4 fields/section were captured). ***p < 0.0005; n.s., not significant. (**b**) Growth of B16 melanoma tumor produced in *Rag1*^−/−^ mice transplanted with WT or *Pld2*^−/−^ CD8^+^ T cells together with WT CD4^+^ T cells was analyzed. Weight of the tumors dissected at day 16 were measured and shown as means ± SEM in the left graph (n = 6). *p < 0.05. Shown in the right images are tumors dissected at day 16.

Helper CD4^+^ and cytotoxic CD8^+^ T cells are the major players of adaptive immunity. Since they are recruited into tumor microenvironment to eliminate tumor cells by inducing tumor cell apoptosis^20^, we analyzed the infiltration of these lymphocytes into B16 melanoma tumors. As shown in Fig. 4a, number of CD8^+^ T cells, but not that of CD4^+^ T cells, in the tumor formed in *Pld2*^−/−^ mice was lower than that in the tumor in WT mice, which was confirmed with *Pld2*^−/−^*/*Cas9 mice (Supplementary Fig. S4). Similarly, number of CD8^+^ T cells but not CD4^+^ T cells in the LLC tumor formed in *Pld2*^−/−^ mice was decreased (Supplementary Fig. S5). These results suggest that *Pld2*^−/−^ mice are defective for development and/or infiltration of CD8^+^ T cells. To confirm this idea, we examined whether ablation of *Pld2* in CD8^+^ T cells is responsible for enhanced tumor growth. WT or *Pld2*^−/−^ CD8^+^ T cells were transferred with WT CD4^+^ cells into *Rag1*^−/−^ mice, which lack all mature lymphocytes^21^, then B16 melanoma cells were implanted into those mice. Tumor growth was significantly greater in the mice received *Pld2*^−/−^ CD8^+^ T cells than in the mice received WT CD8^+^ T cells (Fig. 4b). Taken together, these results suggest that PLD2 in CD8^+^ T cells plays a pivotal role in tumor growth suppression.

### Ablation of *Pld2* reduces the number of CD8^+^ T cells in the spleen

**Figure 5:**
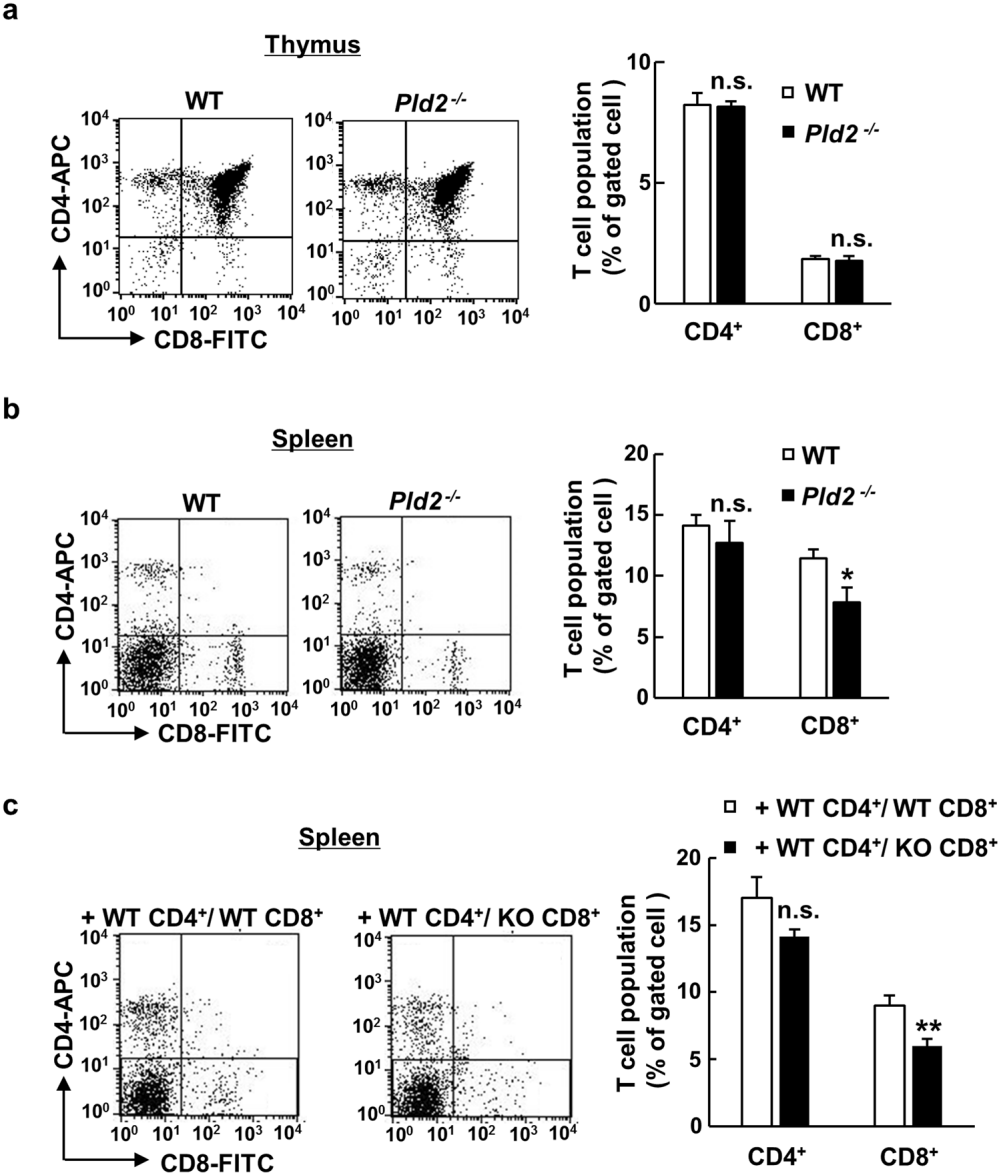
Number of CD8^+^ T cells decreases in the spleen of *Pld2*^−/−^ mice. (**a**,**b**) Thymocytes (**a**) and splenocytes (**b**) isolated from tumor-bearing mice were stained with anti-CD4 and anti-CD8 antibodies, and subjected to FACS analysis. Alive lymphocytes were gated and total of 5 × 10^4^ cells were analyzed (left panels). Data shown are representative of two independent experiments (n ≥ 5 for each experiment). Populations of CD4^+^ and CD8^+^ cells were calculated and shown as means ± SEM in the right graphs. *p < 0.05; n.s., not significant. (**c**) Populations of CD4^+^ and CD8^+^ T cells in the spleens of tumor-bearing *Rag1*^−/−^ mice, in which T cells were transplanted as in Fig. 4b, were analyzed with the FACS system (left panels), and shown as means ± SEM in the right graph (n = 6). **p < 0.01; n.s., not significant.

The developmental process of T lymphocytes includes several steps. Bone marrow-derived T lymphocytes first differentiate in the thymus: immature thymocytes, so called double negative CD4^−^/CD8^−^ cells, differentiate into single-positive CD8^+^ or CD4^+^ naïve T cells through the positive and negative selection^22^. Naïve T cells then migrate to the secondary lymphoid organs such as the lymph node and spleen, where they are activated by antigen presenting cells, followed by clonal expansion and differentiation into effector/memory cells. These activated T lymphocytes migrate throughout the body to eventually reach to and attack infected or damaged tissues. When these T cells in the thymus and spleen were analyzed by flow cytometry, there was no obvious difference in the population of CD4^+^ and CD8^+^ thymocytes between WT and *Pld2*^−/−^ tumor-bearing mice (Fig. 5a). On the other hand, a significant reduction in the population of CD8^+^ T cells was observed in *Pld2*^−/−^ splenocytes (11.46 ± 2.07% versus 7.83 ± 3.44%), while CD4^+^ T cell population unchanged (Fig. 5b). *Rag1*^−/−^ mice transferred with *Pld2*^−/−^ CD8^+^ and WT CD4^+^ T cells also exhibited decreased population of CD8^+^ T cells in the spleen compared to *Rag1*^−/−^ mice transferred with WT CD8^+^ and WT CD4^+^ T cells (Fig. 5c), confirming that ablation of *Pld2* in CD8^+^ T cells is responsible for their reduced population in the spleen. These results raise a possibility that migration of naïve CD8^+^ T cells from the thymus to the spleen or their proliferation/expansion in the spleen is disrupted by the deletion of *Pld2* in CD8^+^ T cells.

### TCR-stimulated proliferation of CD8^+^ T cells is impaired by deletion of *Pld2*

To clarify the possibility described above, we analyzed the *in vitro* chemotaxis ability of naïve CD8^+^ T cells isolated from the thymus of WT and *Pld2*^−/−^ mice. Chemotaxis of naïve CD8^+^ T cells purified from the *Pld2*^−/−^ thymus towards the chemokines CCL21 and CXCL12, which have been implicated in naïve T lymphocyte trafficking to secondary lymphoid organs^23,24^, was comparable to that of WT CD8^+^ T cells (Supplementary Fig. S6). Thus, PLD2 is dispensable for naïve CD8^+^ T cell migration.

**Figure 6:**
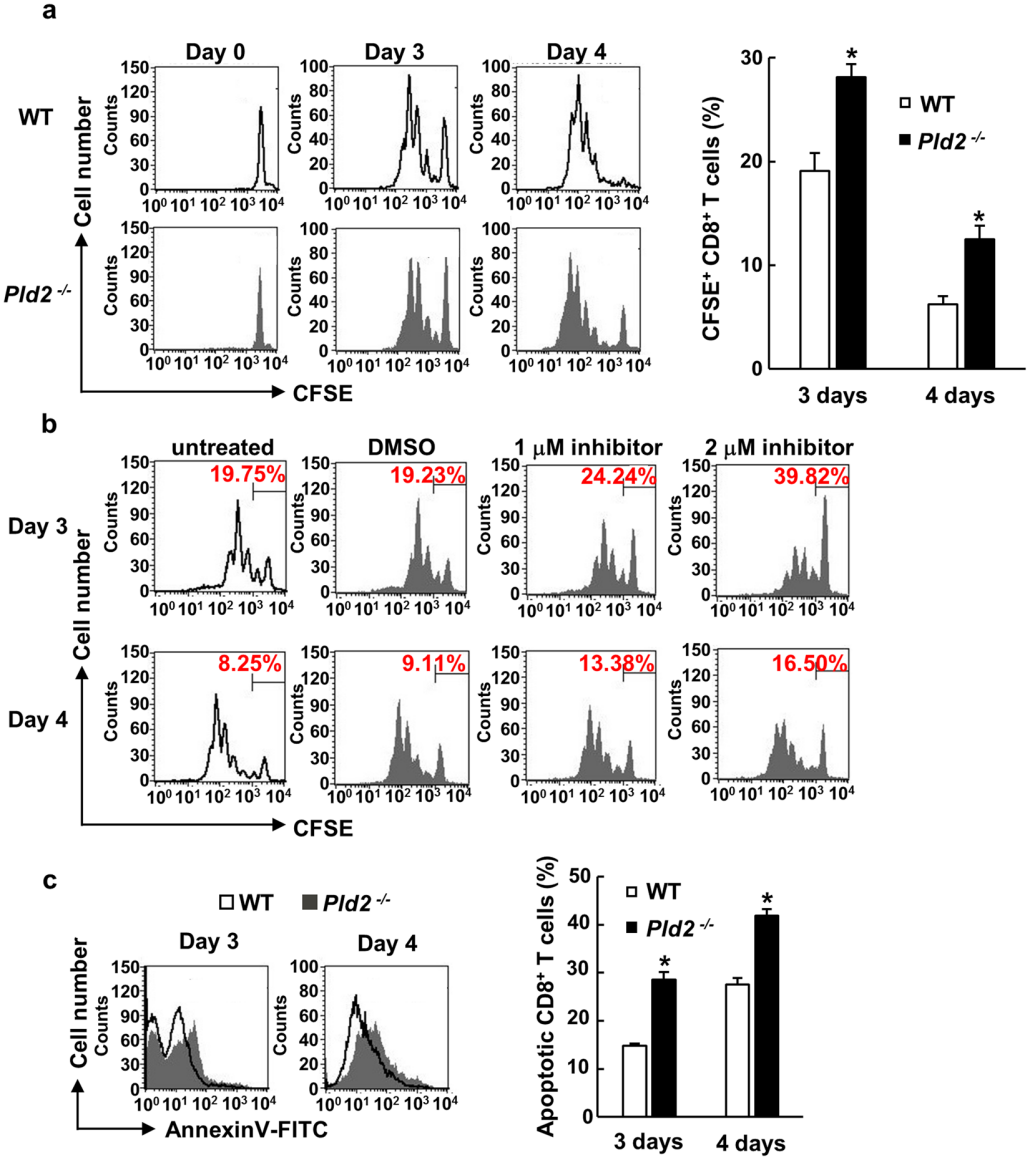
PLD2 is required for proliferation and survival of splenic CD8^+^ T cells. (**a**) Splenic CD8^+^ T cells isolated from WT and *Pld2*^−/−^ mice were labeled with CFSE, and stimulated with immobilized anti-CD3 and soluble anti-CD28 antibody for the indicated days. Proliferation of alive WT (upper panels) and *Pld2*^−/−^ CD8^+^ T cells (lower panels) was analyzed by measuring CFSE fluorescence with the FACS system (left panels). Percentage of CFSE-positive CD8^+^ T cells is shown in the right graph as means ± SEM from at least three independent experiments (n = 6 for each group). *p < 0.05. (**b**) Splenic CD8^+^ T cells isolated from WT mice were treated with or without the PLD2 inhibitor VU0364739, and their proliferation was analyzed as in (**a**). DMSO was used as a control. The number in each panel represents the percentage of CFSE-positive cells. (**c**) Splenic CD8^+^ T cells were isolated and stimulated as in (**a**). The proportion of apoptotic CD8^+^ T cells was evaluated by Annexin V staining and FACS analysis (left panels). Shown in FACS graphs are the representatives of three independent experiments (n = 9 for each group). Quantified data are expressed as means ± SEM. *p < 0.05.

Next, we analyzed the effects of *Pld2* ablation on proliferation of splenic CD8^+^ T cells stimulated with anti-CD3 and -CD28 antibodies, which mimic stimulation by antigen-presenting cells, by the Carboxyfluorescein diacetate succinimidyl ester (CFSE) fluorescence intensity method^25^. Purification of CD8^+^ T cells was more than 95% (Supplementary Fig. S7). CD3/CD28 stimulation by antibodies increased the PLD enzymatic activity in CD8^+^ T cells, which was almost completely suppressed by the deletion of *Pld2* (Supplementary Fig. S8), indicating that PLD2 but not PLD1 is activated by CD3/CD28 stimulation to play a role in TCR-mediated cellular functions. CD3/CD28-stimulated decrease in CFSE intensities of CD8^+^ T cells with prolonged period of culture was slower in *Pld2*^−/−^ CD8^+^ T cells (Fig. 6a), demonstrating that their proliferation is impaired by the *Pld2* ablation. Treatment of WT CD8^+^ T cells with the PLD2-specific inhibitor VU0364739^26^ also suppressed their proliferation (Fig. 6b), confirming that PLD2 is required for CD3/CD28-stimulated CD8^+^ T cell proliferation. On the other hand, any significant difference in the proliferation of WT and *Pld2*^−/−^ CD4^+^ T cells was not observed (Supplementary Fig. S9). It was also found that apoptosis was promoted in *Pld2*^−/−^ CD8^+^ T cells stimulated with anti-CD3 and -CD28 antibodies (Fig. 6c). Thus, PLD2 might be required for proliferation and survival of CD8^+^ T cells, which was supported by the result showing that expression levels of the PLD2 protein and mRNA in CD8^+^ T cells are higher than those in CD4^+^ T cells (Supplementary Fig. S10).

The inhibition of CD8^+^ T cell proliferation by *Pld2* ablation may be resulting from the impaired CD8^+^ T cell activation. This possibility is unlikely because the ratio of the early activation markers CD25- and CD69-positive cells after stimulation of CD3/CD28 were both increased to the comparable levels in WT and *Pld2*^−/−^ CD8^+^ T cells (Supplementary Fig. S11).

### PLD2 is involved in Ras/Erk signaling pathway in CD8^+^ T cells

**Figure 7:**
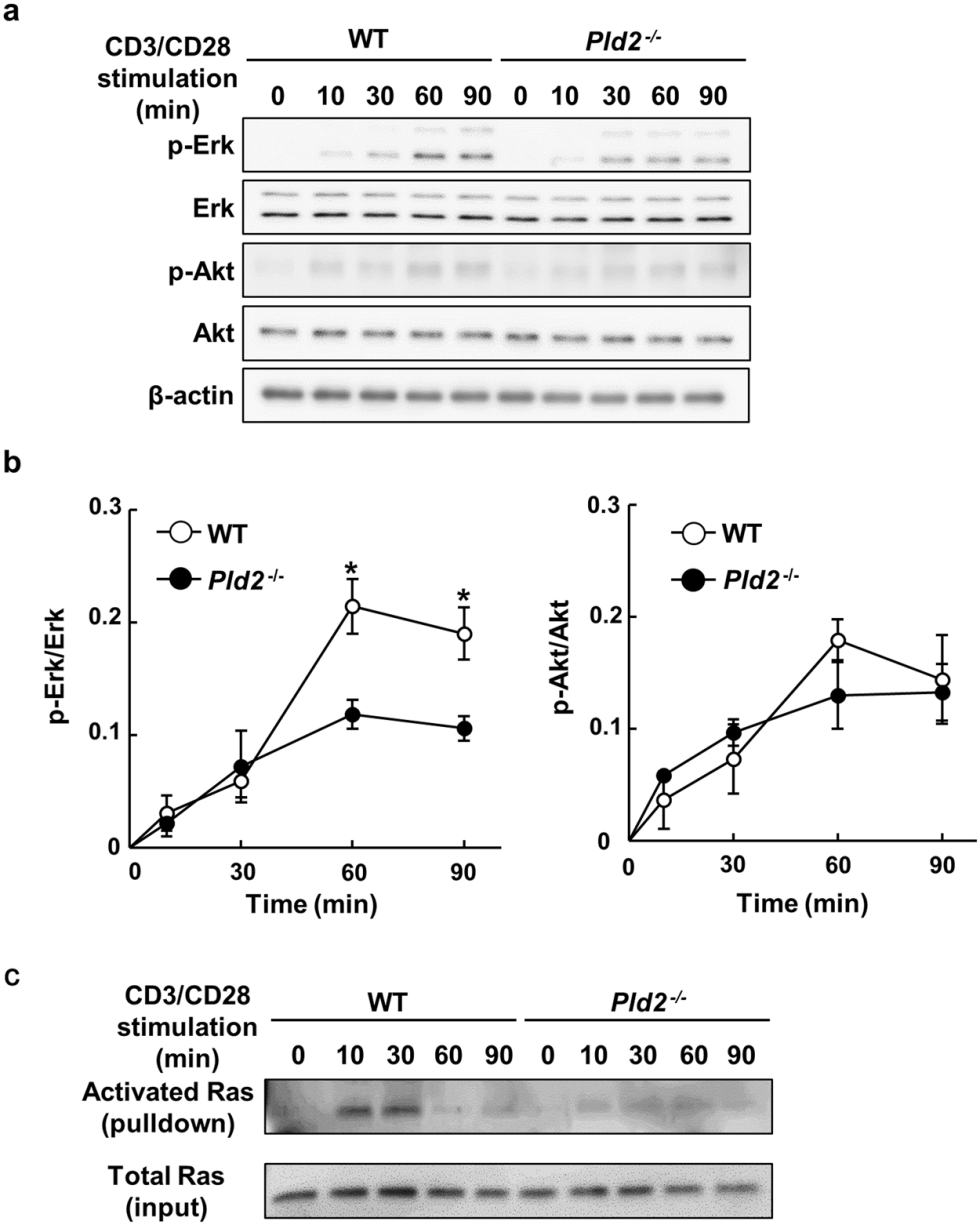
PLD2 is involved in activation of Ras and Erk in CD3-/CD28-costimulated CD8^+^ T cells. (**a**) Primary splenic CD8^+^ T cells were stimulated as in Fig. 6a for indicated time, and phosphorylation of Erk and Akt was analyzed by Western blotting. (**b**) Phosphorylation levels of Erk (left) and Akt (right) in (**a**) were quantitated. Shown are means ± SEM from three independent experiments (n = 6 for each genotype). *p < 0.05. (**c**) Splenic CD8^+^ T cells were stimulated as in (**a**) and activation of Ras was assessed by the pull-down assay using the Ras activation assay kit (Millipore). Shown are representative of two independent experiments.

It is well known that engagement of the TCR activates the PI3K-Akt and Ras-Erk pathways which are critical for the T cell proliferation^27,28^. To elucidate the molecular mechanism how PLD2 regulates CD8^+^ T cell proliferation, we examined the involvement of PLD2 in these pathways. In CD8^+^ T cells isolated from spleens of *Pld2*^−/−^ mice, CD3/CD28-stimulated Erk phosphorylation was significantly suppressed, whereas Akt phosphorylation was unaffected by *Pld2* ablation (Fig. 7a,b). Furthermore, activation of Ras, the upstream activator of the Erk pathway, was inhibited in *Pld2*^−/−^ CD8^+^ T cells (Fig. 7c). Production of interleukin-2 (IL-2), which is the downstream target gene product of Erk and the extracellular signaling molecule for CD8^+^ T cell proliferation, decreased in *Pld2*^−/−^ CD8^+^ T cells (Supplementary Fig. S12). In contrast, production of Interferon-γ (IFN-γ) and tumor necrosis factor-α (TNF-α), both of which are secreted from effector CD8^+^ T cells to resist against pathogens, was not affected by *Pld2* ablation (Supplementary Fig. S12). The inhibition of Ras/Erk activation and IL-2 production is unlikely to be attributable to the downregulation of TCR or CD28: the cell surface levels of TCR and CD28 during the CD3/CD28 stimulation were comparable between WT and *Pld2*^−/−^ CD8^+^ T cells (Supplementary Fig. S13). Finally, pharmacological inhibition of MEK, the upstream kinase of Erk, by the inhibitor U0126 demonstrated that suppression of Erk phosphorylation is sufficient to inhibit CD3/CD28-stimulated proliferation of splenic CD8^+^ T cells (Supplementary Fig. S14). Taken together, PLD2 appears to be required for TCR-mediated activation of the Ras-Erk pathway and IL-2 production, which are essential cell events for TCR-stimulated proliferation of splenic CD8^+^ T cells.

## Discussion

The results obtained in this study provide evidence that PLD2 in CD8^+^ T lymphocytes plays an important role in the TCR-mediated proliferation of these cells in the spleen by regulating the Ras-Erk signaling pathway and IL-2 production, thereby at least in part contributing to the anti-tumor immunity. We began this study with *Pld2*^−/−^ mice, which have previously been generated^17^, and found that *Pld2* deletion promoted tumor growth. In this result, it was our concern that a partial peptide is produced in the *Pld2*^−/−^ mice, which have been generated by replacing exons 14 and 15 of the *Pld2* gene, and exerts an off-target effect(s). However, this is not likely because the new *Pld2*-null mice, *Pld2*^−/−^*/*Cas9, which were generated by deleting most of coding regions of the *Pld2* gene, showed the same phenotype. Thus, it can be concluded that PLD2 in CD8^+^ T cells plays an important role in the anti-tumor immunity.

Infiltration of leukocytes into tumor environment is a hallmark of anti-cancer immunity: tumor-associated macrophages and T cells are frequently found^5,29^. It has been shown that the mice transplanted with CD8^+^ T cells acquire both immediate and long-term protection from tumors: 93% and 30–60% of mice transplanted with CD8^+^ T cells were tumor-free when they were challenged with tumors after 1 day and 90 days after transplantation, respectively^30^. In addition, Sato *et al*. have reported that among tumor-infiltrating lymphocytes (TILs), only intraepithelial CD8^+^ TILs were associated with improved survival of epithelial ovarian cancer patients^31^. These reports provide evidence for the central role of CD8^+^ T cells in anti-tumor immunity. Furthermore, CD8^+^ T cells eliminate target cells including tumor cells by inducing apoptosis^32^, and in this study we observed decreased CD8^+^ T cell infiltration in tumors formed in *Pld2*^−/−^ mice concomitantly with the reduced number of apoptotic tumor cells. These lines of evidence are consistent with the notion that impaired CD8^+^ T cell infiltration into tumor microenvironment in *Pld2*^−/−^ mice promotes tumor growth.

In the present study, we focused on the function of PLD2 in CD4^+^ and CD8^+^ T cells to elucidate the pathological function of PLD2 in tumor growth. However, we cannot still rule out the possibility that PLD2 in other immune cells is also involved in suppression of tumor growth. Several lines of evidence demonstrate that PLD2 regulates functions of some immune cells distinct from T lymphocytes. In a macrophage cell line RAW/LR5, PLD2 participates in cell chemotaxis^33^, and is required for phagocytosis of both macrophages and neutrophils^12,34^. Furthermore, deficiency of *Pld2* impairs the migration and extravasation of primary neutrophils^12^. These reports indicate the possibility that PLD2 contributes to anti-tumor immunity through these immune cell functions. It is also of great interest to elucidate whether PLD2 regulates functions of regulatory T (Treg) cells, which suppress T cell-mediated immune responses^35^. If PLD2 negatively regulates the effector T cell-suppressive function of Treg cells, ablation of *Pld2* from Treg cells would interfere with the anti-tumor immune response of CD8^+^ T cells. Since inhibition of Treg cell function strongly suppresses tumor progression^36,37^, clarification of the PLD2 involvement in the function of Treg cells might provide insight into anti-cancer immunotherapies.

Although we showed that PLD2 is critical for proliferation and survival of activated CD8^+^ T cells, thereby contributing to suppression of tumor growth, PLD2 in other types of cells distinct from immune cells might be also involved in the suppression of tumor growth. This notion is derived from the observations that tumor growth in WT mice transplanted with *Pld2*^−/−^ bone marrow cells was slower than that in *Pld2*^−/−^ mice transplanted with *Pld2*^−/−^ bone marrow cells, and that tumor growth in *Pld2*^−/−^ mice transplanted with WT bone marrow cells tended to be stimulated compared with that in WT mice transplanted with WT bone marrow cells (Fig. 3b). It is known that the interaction between tumor cells and stromal cells is critical in tumor progression: both types of cells mutually support their proliferation by secreting and recognizing growth factors, such as epidermal growth factor (EGF), in an autocrine/paracrine manner, thereby promoting tumor growth^38^. Since PLD2 has been reported to promote the EGF receptor (EGFR) endocytosis through the activation of the Dynamin GTPase^39^, PLD2 might inhibit the EGF/EGFR circuit among tumor cells and stromal cells by limiting cell surface EGFR of stromal cells.

Recently, Ghim *et al*. have reported that tumor angiogenesis is inhibited in endothelial cell-specific *Pld2* conditional knockout mice^40^, demonstrating that PLD2 in vascular endothelial cells is essential for tumor angiogenesis. On the other hand, we did not observe any changes in tumor angiogenesis in *Pld2*^−/−^ mice (Fig. 2a), even though PLD2 could be deleted from vascular endothelial cells in these mice. Although we cannot clearly explain why these two studies showed different phenotypes, it is possible that pro-angiogenic and anti-angiogenic functions of vascular endothelial cells and other cell types, respectively, are balanced in *Pld2*^−/−^ mice; therefore, *Pld2*^−/−^ mice did not exhibit apparent changes in tumor angiogenesis. It is also plausible that PLD2 in some types of cells in tumor microenvironment is involved in inhibition of pro-angiogenic factor production/secretion. Further studies are required to clarify these issues.

In the dissection of the signaling pathways for the PLD2-mediated T cell proliferation, we found that PLD2 activates Ras and subsequently promotes phosphorylation of Erk1/2 in the CD3/CD28-stimulated primary cultured CD8^+^ T cells. However, it still remains to clarify the activation mechanism of Ras by PLD2. Mor *et al*. have suggested that PLD2 activates Ras through the action of RasGRP1, the guanine nucleotide-exchange factor (GEF) of Ras, at the plasma membrane in Jurkat cells co-stimulated with TCR and lymphocyte function-associated antigen-1 (LFA-1)^41^. Furthermore, Salzer *et al*. have recently indicated that RasGRP1-deficient CD8^+^ T cells prepared from immunodeficient patients show reduced Erk1/2 phosphorylation^42^. From these reports, it is reasonable to speculate that upon TCR engagement of CD8^+^ T cells, PLD2 recruits the Ras GEF RasGRP1 to the plasma membrane to activate Ras, which in turn activates Erk1/2 to promote cell proliferation and survival. A distinct Ras GEF might also be involved in Ras activation, because Son of sevenless 1 (Sos1), another Ras GEF, has been reported to be involved in T cell proliferation by persistently activating Erk downstream of TCR^43^. Interestingly, PA directly binds to the PH domain of Sos1 and recruits it to the plasma membrane to activate Ras^44^. Since TCR-stimulated PLD enzymatic activity in CD8^+^ T cells mostly depends on PLD2 (Supplementary Fig. S7), it is plausible that PLD2 activates Ras by recruiting Sos1 to the plasma membrane through its product PA. The detailed mechanism for the PLD2 activation of Ras remains to be elucidated by the future analysis.

Although we showed that PLD2 regulates CD8^+^ T cell proliferation through the Ras-Erk pathway, involvement of an additional pathway(s) cannot be excluded. Several lines of evidence demonstrate that c-Jun amino terminal kinase (JNK), another member of the MAP kinase family, is involved in the regulation of CD8^+^ T cell functions. Conze *et al*. (2002) reported that the absence of JNK2 increases IL-2 production and proliferation of CD8^+^ T cells. On the other hand, CD8^+^ T cells lacking JNK1 are unable to undergo antigen-stimulated expansion *in vitro*^45^. In addition, Gao *et al*. (2005) indicated that deletion of *Jnk1* from CD8^+^ T lymphocytes reduces perforin expression and impaired CTL functions, which are responsible for tumor susceptibility^46^. It is of interest to examine whether JNK is regulated by PLD2 in TCR-stimulated CD8^+^ T cells.

Interestingly, we found that PLD2 in CD8^+^ T cells is involved in production of IL-2, which regulates multiple immune response-related functions of CD8^+^ T cells, including clonal expansion/proliferation, memory generation, cytotoxicity, and IFN-γ production^47,48^. The study on *Il-2*-deficient mice showed that although CD8^+^ T cells can be weakly activated and elicit cytotoxicity during infection in an IL-2-independent mechanism, infection-dependent cell expansion exclusively requires IL-2, suggesting that IL-2 plays a unique role in proliferative responses of CD8^+^ T cell^48^. Since IL-2 produced in and secreted from CD8^+^ T cells contributes to proliferation of CD8^+^ T cells in an autocrine/paracrine way, our findings indicate that IL-2 is the one of the major target genes for the PLD2-Ras-Erk axis to promote CD8^+^ T cell proliferation.

In this study, we used two different cancer cell lines, B16 melanoma and Lewis lung carcinoma, to induce tumor formation in mice. From the unexpected observation of tumor growth in two independent knockout mice models, we uncovered the novel function of PLD2 in CD8^+^ T cell proliferation. Since CD8^+^ T cells eliminate various types of cancer, it is of interest to examine progression of other types of cancer in *Pld2*-deleted mice.

Recent advances in understanding anti-tumor immunity have provided insight into new approaches for cancer treatment. The results in this study provide insight into the mechanism governing anti-tumor immunity and could contribute to improve cancer immunotherapy. Screening of chemical libraries to activate PLD2 or creation of membrane permeable PA could be a new strategy for the treatment of cancers and immunodeficiency disorders.

## Materials and Methods

### Mice

*Pld2*^−/−^ mice with C57BL/6 background were described previously^17^. *Pld2*^−/−^*/Cas9* mice were generated as described in the report by Mizuno *et al*.^49^, and a schematic representation of *Pld2* targeting is shown in Supplementary Fig. S1. Briefly, we determined the candidate CRISPR target sequences of the *Pld2* gene using the website CRIPR direct (https://crispr.dbcls.jp/). Oligo DNAs for the respective target sequences were designed and inserted into the px300 vector (Addgene plasmid 42230) to generate px300-*Pld2*, which expresses gRNA for the *Pld2* gene and the Cas9 protein. The cleavage efficiencies of the target sequences by these vectors were evaluated by the pCAG-EGxxFP (Addgene plasmid 50716) system^49^. The gRNA for position 163–185 of the *Pld2* gene was selected according to its highest efficiency. The donor oligo DNA containing stop codons in three different frames was designed for homology-directed repair (HDR)-mediated gene modification (Supplementary Fig. S1). The px300-*Pld2* and the oligo donor were injected into pronuclei of one-cell-stage embryos collected from C57BL/6 mice. The injected embryos were then transferred into pseudopregnant ICR mice. Targeted mice were screened by PCR genotyping and direct sequencing of the tail DNA genome using the primers of 5′-GCCATCTATGACCTTCAGCCTCTGAAAGC-3′ and 5′-GTCTTCTGGCCCAACCCAGGCCTCCACTT-3′. Age- and sex-matched 8 to 12 weeks-old mice were used in the experiments. All experiments with mice were conducted according to the Guidelines for Proper Conduct of Animal Experiments, Science Council of Japan, and protocols were approved by the Animal Care and Use Committee, University of Tsukuba.

### Cell culture

B16 melanoma cells with or without stable expression of mCherry and Lewis lung carcinoma (LLC) cells were maintained in Dulbecco’s modified Eagle’s medium (DMEM) (Nacalai Tesque) containing 10% fetal bovine serum (FBS; Gibco) and 1% penicillin-streptomycine (P/S) (Nacalai Tesque). Primary CD4^+^ T cells and CD8^+^ T cells were cultured in RPMI1640 (Nacalai Tesque) supplemented with 10% FBS, 50 μM 2-mercaptoetanol and 2% P/S at 37 °C under 5% CO_2_.

### Assay for tumor growth

B16 melanoma (10^6^ cells) or LLC cells (2.5 × 10^5^ cells) suspended in 100 μl of serum-free DMEM were subcutaneously transplanted into the dorsal flank of 8 weeks-old mice. Tumor volume was measured by digital caliper every other day after sixth day of implantation, and calculated according to the following formula: tumor volume = length × (width)^2^ × 0.52. After 16 or 18 days of implantation, tumors were dissected, fixed in 4% FPA/PBS at 4 °C for 4 hr, followed by incubation in 30% sucrose at 4 °C overnight, and subjected to immunohistochemistry analyses.

### Immunohistochemistry

Tumors were sliced into 7 μm-thick sections, fixed with 4% paraformaldehyde/PBS for 10 min at room temperature (RT), and blocked with TNB buffer [0.1 M Tris-HCl, pH 7.5, 0.15 M NaCl, 0.5% TSA blocking reagent (PerkinElmer)] for 1 hr at RT. To analyze tumor angiogenesis, sections were incubated with anti-PECAM1 (1:200 dilution; Biolegend) and -α-SMA (1:200 dilution; Abcam ab5649) antibodies at 4 °C overnight. To examine tumor-infiltrated T cells, sections were incubated with primary antibodies against CD4 (clone GK1.5) and CD8 (clone 53–6.7) (1:200 dilution; Biolegend) at 4 °C overnight, and subsequently with Alexa 488-conjugated secondary antibodies (1:1000 dilution; Invitrogen). Fluorescence signals were observed using the fluorescence microscope Biozero (KEYENCE).

### Assay for apoptosis

Cell apoptosis was analyzed by the TUNEL assay and immunostaining of cleaved caspase-3. TUNEL assay was performed using the *In Situ* Cell Death Detection Kit Fuorescein (Roche) according to the manufacturer’s instruction. In brief, tumor sections were fixed with 4% FPA/PBS for 15 min at RT and permeabilized with 0.1% Triton X/0.1% sodium citrate for 5 min on ice. After washing with PBS/0.1% BSA, sections were labeled with the TUNEL reaction mixture at 37 °C in the dark for 60 min. For the detection of cleaved caspase-3, tumor sections were immunostained with the anti-cleaved caspase-3 antibody (Cell Signaling, #9661) as described above. The number of apoptotic cells and B16 melanoma-apoptotic cells were evaluated by capturing both the peripheral and central fields of each section (at least 4 fields/section).

### Flow cytometry

Splenocytes and thymocytes were isolated from the spleen and thymus, respectively. Red blood cells were lysed with Tris-buffered ammonium chloride (ACTB) and remaining cells were sequentially filtered through 70 μm and 45 μm membranes to remove cell aggregates. Cells were blocked with anti-mouse CD16/32 antibodies (Biolegend), and stained with FITC-conjugated anti-CD8, APC-conjugated anti-CD4 antibodies (Biolegend), and propidium iodide (PI), which stains dead cells. Flow cytometry was performed with the BD FACSCalibur cytometer (BD Bioscience) and data were analyzed using the BioCell Quest software.

### Isolation of primary T cells

Primary CD4^+^ and CD8^+^ T cells were purified from splenocytes and thymocytes by negative selection. In brief, splenocytes and thymocytes were incubated with the biotinylated antibody cocktail including 0.5 µg/10^6^ cells of anti-B220, -CD19, -IgM, -MHCII, -CD49b, -CD16/32, -Cd11c, -CD11b, -Ly-6G/6 C, -CD24, and -CD4 or -CD8 antibodies (Biolegend) at 4 °C for 30 min: the cocktail contained anti-CD4 or -CD8 antibody for CD8^+^ or CD4^+^ T cell purification. Dynabeads MyOne Streptavidin C1 (Invitrogen) was added to the cell suspension and incubated with rotation for 30 min at 4 °C. Cell suspension was placed in a magnet for 2 min to remove bead-bound cells and the supernatant which contains target cells was collected. Isolated T cells were incubated for 3–4 hr in RPMI 1640 containing 10% FBS and 2% P/S at 37 °C, 5% CO_2_, then subjected to assays for cell migration and *in vitro* proliferation.

### Cell migration assay

Primary CD8^+^ T cells suspended in RPMI1640 were placed in the upper chamber of trans-well migration chambers (5 μm pore size; Corning). The lower chamber was filled with RPMI1640/0.1% BSA supplemented with or without 50 ng/ml of the chemokine, recombinant murine SDF-1α (CXCL12) (Peprotech) or recombinant murine Exodus-2 (CCL21) (Peprotech). After 3 hr of incubation, migrated cells in the lower chamber were collected by centrifuge at 2,000 × g for 10 min, and counted with haemocytometer. Migrated cells were expressed as % of total initial cells in the upper chamber.

### Bone marrow transplantation

Recipient WT or *Pld2*^−/−^ mice were irradiated with X-ray (Hitachi MBR-1520R-3) at 4.5 Gγ twice at an interval of 4 hr. Bone marrow cells were isolated from donor WT or *Pld2*^−/−^ mice, and 5 × 10^6^ cells suspended in PBS were intravenously injected into irradiated recipient mice after 24 hr of irradiation. At 12 weeks after irradiation, tumor cells were implanted.

### T cell transplantation

Either WT or *Pld2*^−/−^ CD8^+^ T cells (5 × 10^6^ cells) and WT CD4^+^ T cells (7 × 10^6^ cells) suspended in PBS were injected i.v. into *Rag1*^−/−^ recipient mice. One week after transplantation, B16 melanoma cells were implanted as described above.

### *In vitro* cell proliferation assay

CD8^+^ T cells (3 × 10^7^ cells) isolated from the spleen of WT or *Pld2*^−/−^ mice were labeled with the fluorescent dye CFSE (3 μM). In the experiments with the PLD2 inhibitor VU0364739 (1 μM or 2 μM; Tocris Bioscience #4171) or the MEK inhibitor U0126 (5 μM; Cell Signaling #9903), cells were treated with inhibitors for 2 hr before labeling with CFSE. Cells were then seeded on the 6-well plate at 3 × 10^6^ cells/well and stimulated with 5 μg/ml of anti-CD3 antibody (clone 17A2; Biolegend) pre-bound to the culture plate and 2 μg/ml of soluble anti-CD28 antibody (clone 37.51; Biolegend) at 37 °C for the indicated days. Proliferation capacity of the cell was analyzed by tracing dilution of CFSE fluorescent intensity by flow cytometry.

### Measurement of PLD activity

CD8^+^ T cells (2 × 10^7^ cells/ml) isolated from the spleen of WT or *Pld2*^−/−^ mice were suspended in RPMI 1640/1% FBS and incubated with [^3^H]lyso-PAF (5 µCi/ ml) at 37 °C for 1 hr to label PC in lymphocytes. Cells were washed with PBS/1% FBS for 3 times, suspended in HBSS/1% FBS plus 1% ethanol at 5 × 10^6^ cells/ml, and co-stimulated with antibodies against CD3 and CD28 as described above for the indicated time. Cell lipids were extracted and separated by thin-layer chromatography on a silica gel plate. Phosphatidylethanol (PEt), the PLD-specific product in the presence of ethanol, was visualized by iodine staining, and the radioactivity of PEt was measured as described previously^17^. PEt production was calculated by dividing the radioactivity of PEt by that of the total phospholipids.

### Assay for cytokine production in CD8^+^ cells

CD8^+^ T cells isolated from the spleen of WT and *Pld2*^−/−^ mice were seeded on 12-well plates at 2 × 10^5^ cells/well and stimulated with anti-CD3/CD28 antibodies for 24 hr as described above. Brefeldin A (eBioscience) was added to the culture for the final 6 hr incubation to block cytokine secretion. Cells were collected and stained for CD8 with anti-CD8 antibody for 30 min at 4 °C, followed by fixation and permeabilization with the Intracellular Fixation and Permeabilization buffer set (eBioscience). After washing, antibodies against IL-2 (clone JES6–5H4; BD Biosciences), IFN-γ (clone XMG1.2; BD Biosciences) and TNF-α (clone MP6-XT22; BD Biosciences) were added to the cells and subjected to flow cytometry analysis, and cell number producing these cytokines was counted.

### Western blotting

To analyze the expression levels of PLD2, mouse brain and isolated primary T lymphocytes were lysed in urea buffer (8 M urea, 50 mM Tris-HCl, pH 6.8, 0.01% SDS, 0.01% β-mercaptoethanol, and 0.001% bromophenol blue) and in lysis buffer (1% Nonidet P-40; 150 mM NaCl; 50 mM Tris with protease inhibitor), respectively. After lysates were sonicated, proteins in the lysate were separated by 10% SDS-polyacrylamide gel electrophoresis, and transferred onto the PDVF membrane. The membrane was blocked with Blocking Buffer (TOYOBO) for 1 hr at RT and incubated with the anti-PLD2 antibody^17^ diluted (1:1000) in the Can get signal kit (TOYOBO) at 4 °C overnight.

For detection of phosphorylated Erk and Akt, CD8^+^ T cells were activated with anti-CD3 and -CD28 antibodies and lysed in the lysis buffer supplemented with phosphatase inhibitors as described above. After separation of proteins of lysates and transfer to PDVF membranes, membranes were incubated at 4 °C overnight with primary antibodies for phospho-p44/42 MAPK (Thr202/Tyr204) (Cell signaling), p44/42 MAPK (Erk1/2) (Cell Signaling), phosho-Akt (T308) (Cell Signaling), and Akt (Cell Signaling) which were diluted in 1% BSA/0.05% Tween 20/PBS (1:1000). Membranes were subsequently incubated with HRP-conjugated secondary antibodies (Cell Signaling) at RT for 1 hr. The signals were detected by the luminescent image analyzer LAS-4000 mini (Fujifilm, Japan) and phosphorylation levels of p-Erk and p-Akt were analyzed by the Multi Gauge software (Fujifilim, Japan).

### Statistical analyses

All experiments were presented as the mean ± SEM. The statistical analysis was performed using an unpaired two tail - Student’s *t*-test. *P*-values < 0.05 were considered to be statistically significant.

## Electronic supplementary material

Supplementary Figures
