# Supplementary material for: Physiological function of phospholipase D2 in anti-tumor immunity: regulation of CD8+ T lymphocyte proliferation
Source: Sci Rep. 2018 Apr 19;8:6283. doi: 10.1038/s41598-018-24512-x (PMC5908902; doi:10.1038/s41598-018-24512-x)
Supplement: Supplementary file 1 — Supplementary Figures [file 41598_2018_24512_MOESM1_ESM.pdf]

Supplementary Figures

Physiological function of phospholipase D2 in anti-tumor immunity: regulation of CD8$^+$ T lymphocyte proliferation

Van Ngo Thai Bich, Tsunaki Hongu, Yuki Miura, Naohiro Katagiri, Norihiko Ohbayashi, Yumi Yamashita-Kanemaru, Akira Shibuya, Yuji Funakoshi and Yasunori Kanaho

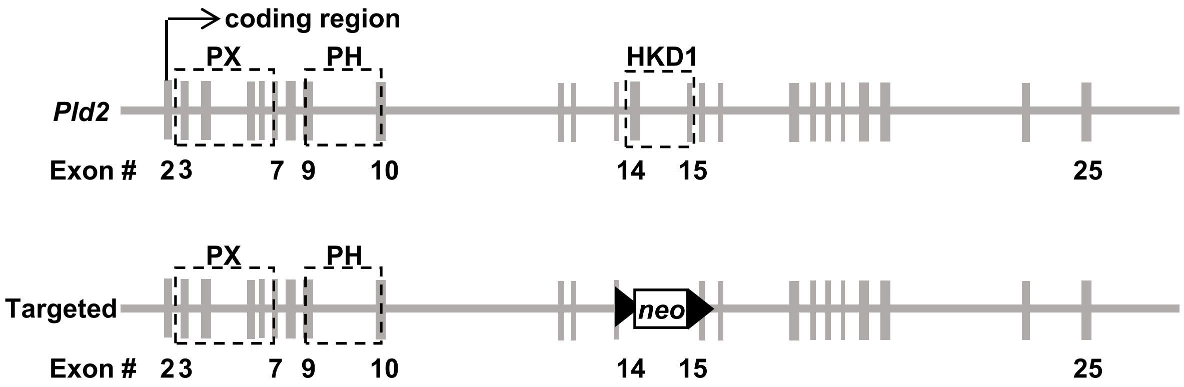

Supplementary Figure S1. Schematic representation of *Pld2* gene. Exons of the *Pld2* gene are represented by filled grey rectangles. Domain structures are represented by dashed boxes. The targeted allele for *Pld2*$^{-/-}$ mice is shown in the bottom. *neo*, neomycin-resistance cassette; filled triangles, *loxP* sites.

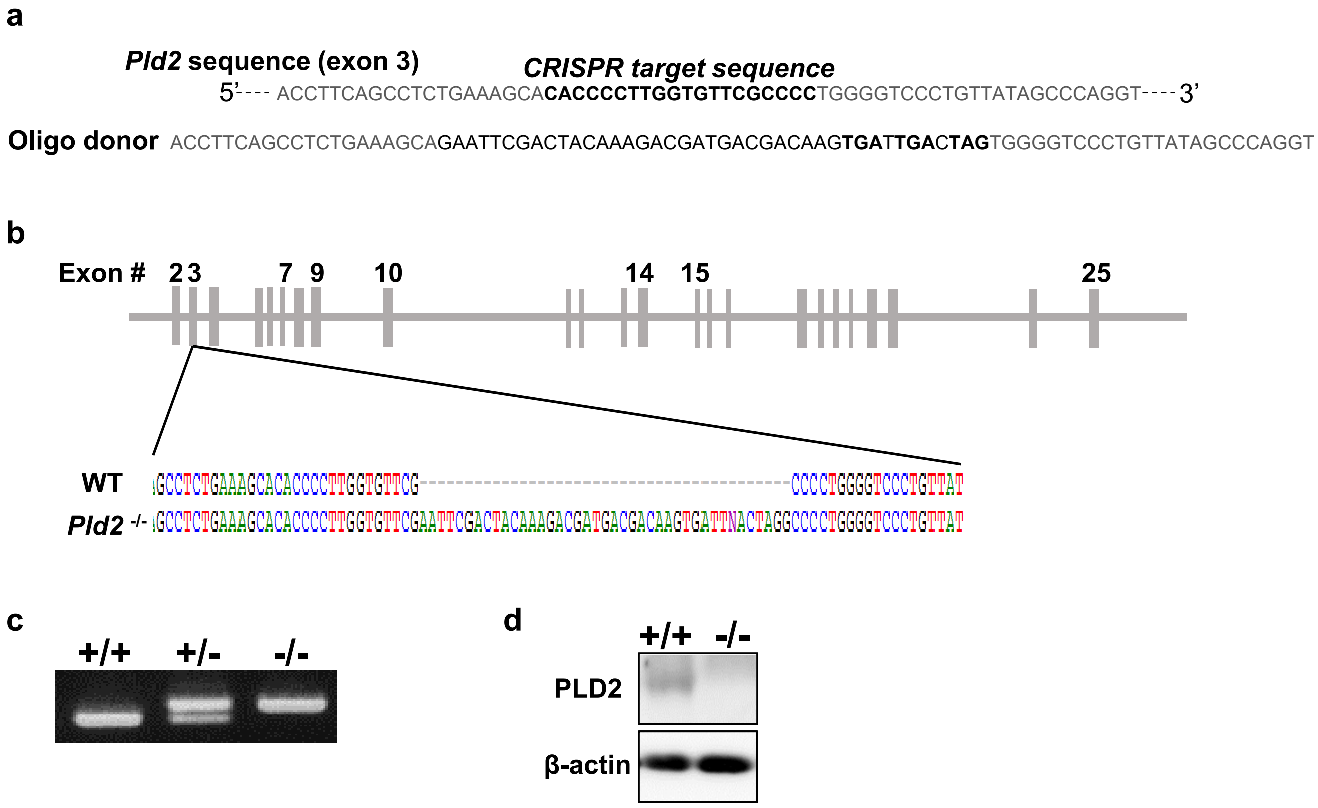

Supplementary Figure S2. Generation of *Pld2*-null mice using the CRISPR/Cas9 system. (a) The CRISPR-Cas9 target site in the exon 3 of the *Pld2* gene (bold letters in the upper sequence) and the 85-nt oligo donor DNA sequence. Three stop codons in the donor DNA sequence are indicated in bold letters. **(b)** Sequence of the targeted site verified by DNA sequencing. (**c**) Genomic PCR analysis for mice of indicated genotypes. (**d**) Western blot for PLD2 in brain lysates of the mice.

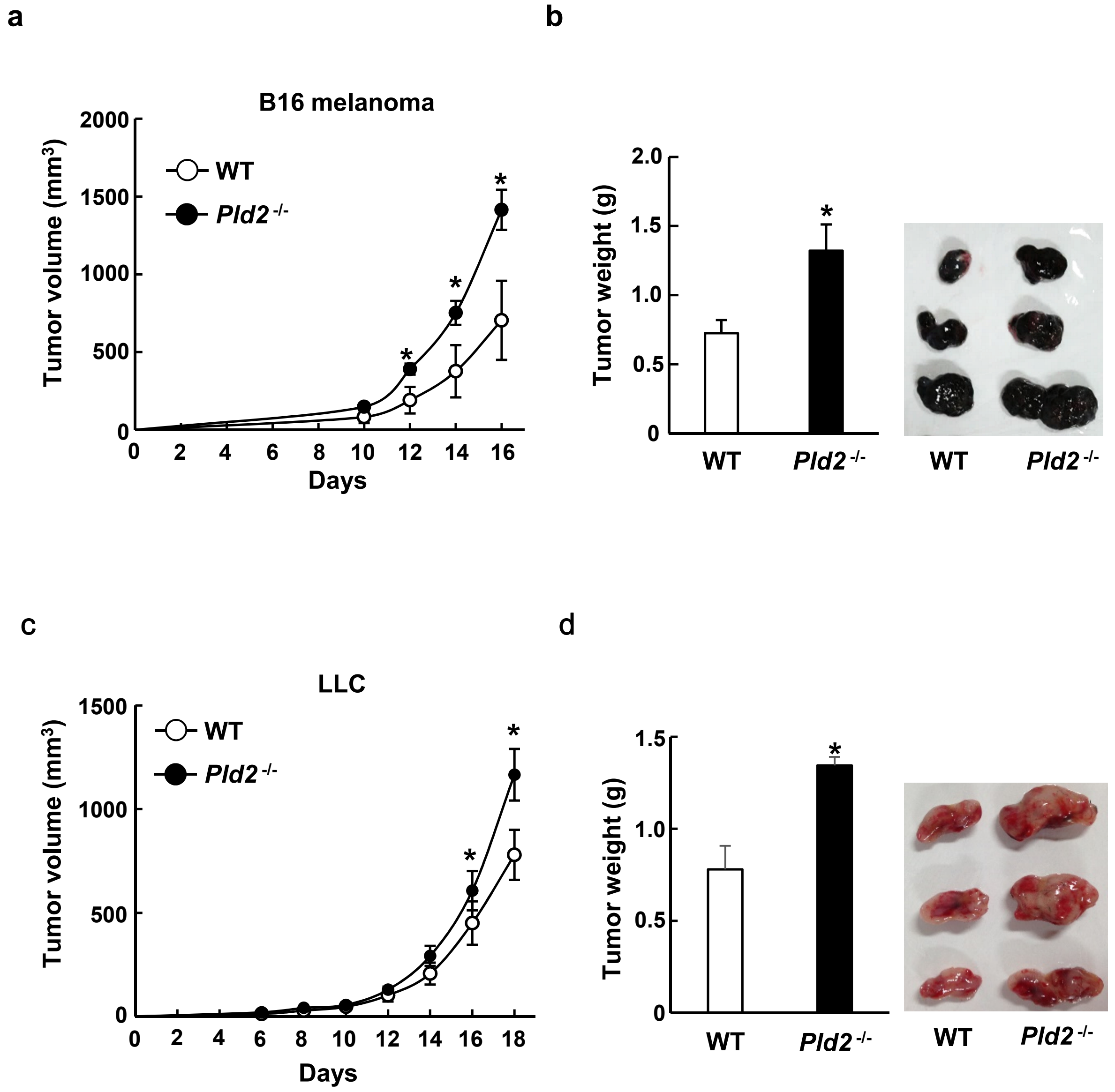

Supplementary Figure S3. Tumor growth is promoted in *Pld2*$^{-/-}$/Cas9 mice. **(a)** Tumor growth produced by implanted B16 melanoma cells in WT (n=7) and *Pld2*$^{-/-}$/Cas9 mice (n=6) was assessed as in Figure 1a. **(b)** Primary tumors were dissected after 16 days of implantation (right panel), and their weight (left panel) were measured and shown as means ± SEM. **(c, d)** Lewis lung carcinoma cells were implanted into WT and *Pld2*$^{-/-}$/Cas9 mice (n=4). Tumor volume and weight were measured as in (a) and (b), respectively. $^*p<0.05$.

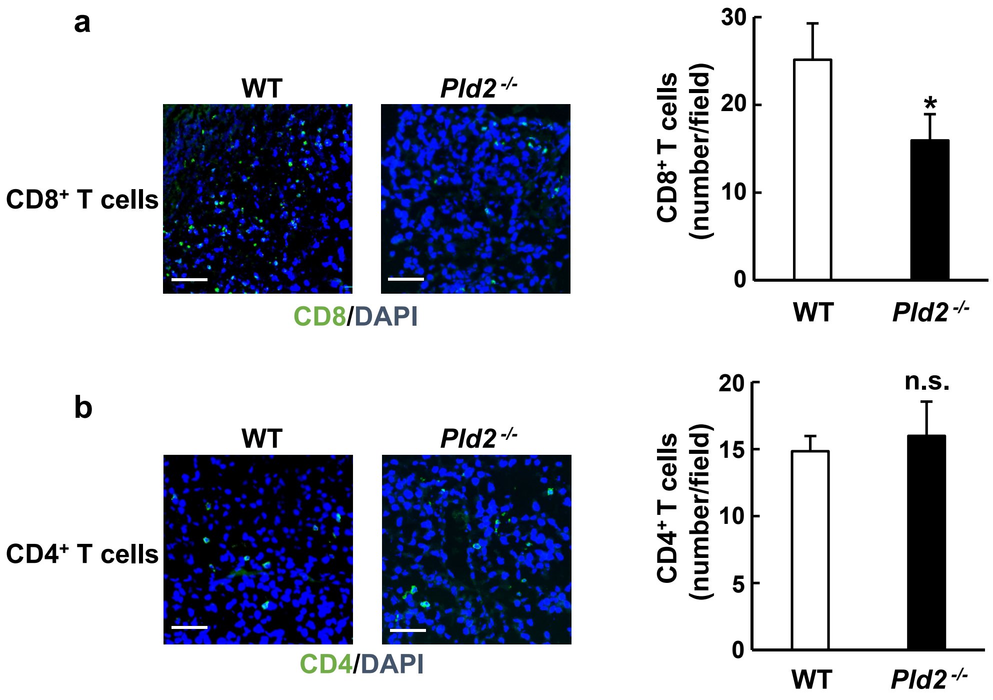

Supplementary Figure S4. Infiltration of CD8⁺ T cells into tumors is suppressed in *Pld2*$^{-/-}$/Cas9 mice. Infiltration of CD8⁺ T cells **(a)** and CD4⁺ T cells **(b)** into tumors was analyzed as in Figure 4a (left panels). Means ± SEM are shown in the right (n=5 for each group, at least 4 fields/section were captured). *p<0.05; n.s., not significant.

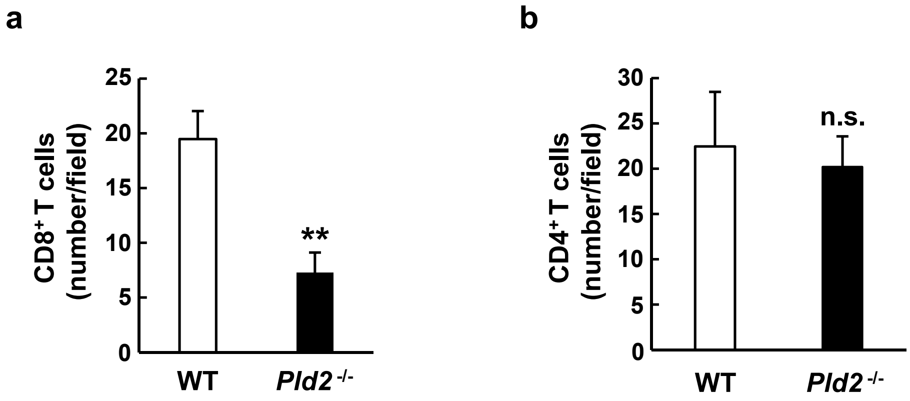

Supplementary Figure S5. Infiltration of $CD8^+$ T cells into Lewis lung carcinoma tumors is impaired in *Pld2*$^{-/-}$ mice. The number of $CD8^+$ T cells **(a)** and $CD4^+$ T cells **(b)** in LLC tumors formed in WT and *Pld2*$^{-/-}$ mice were counted. $**p<0.01$; n.s., not significant.

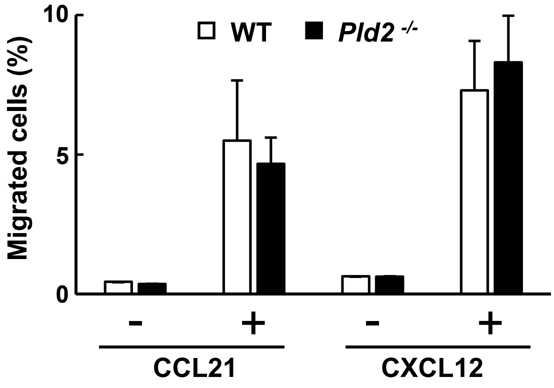

Supplementary Figure S6. Chemotaxis of thymus CD8$^+$ T cells is not affected by *Pld2* deletion. Migration of CD8$^+$ T cells isolated from thymuses of WT and *Pld2*$^{-/-}$ mice toward chemokine CCL21 (50 ng/ml) and CXCL12 (50 ng/ml) was analyzed with a transwell chamber. Shown are means ± SEM from three independent experiments (n=6 for each group).

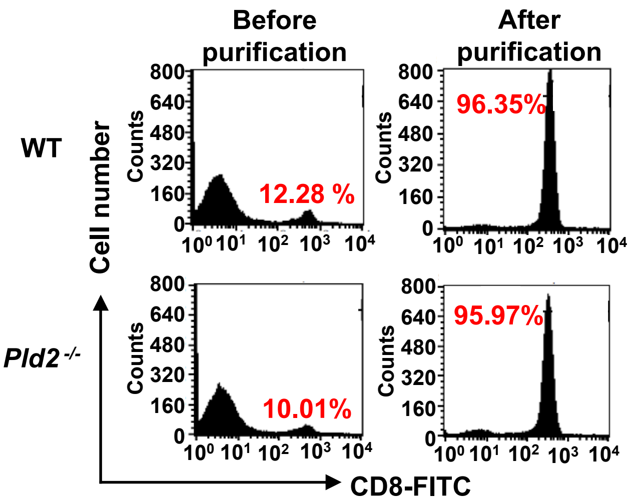

Supplementary Figure S7. Efficiency of CD8^{+} T cell purification. CD8^{+} T cells were purified from the splenocytes by the negative selection and their purity was assessed by FACS.

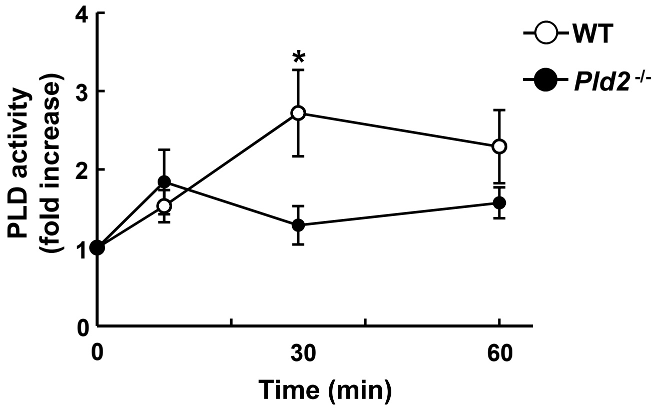

Supplementary Figure S8. PLD2 in CD8$^+$ T cells is activated upon CD3/CD28 stimulation. WT and *Pld2*$^{-/-}$ splenic CD8$^+$ T cells were labeled with [^{3}H]lyso-PAF (5 μCi/ ml) and stimulated with anti-CD3 and -CD28 antibodies in the presence of 1% ethanol for the indicated time. PLD activity was assessed by measuring the produced [^{3}H]PEt. Data are shown as means ± SEM from three independent experiments (n=9 for each group). *$p<0.05$.

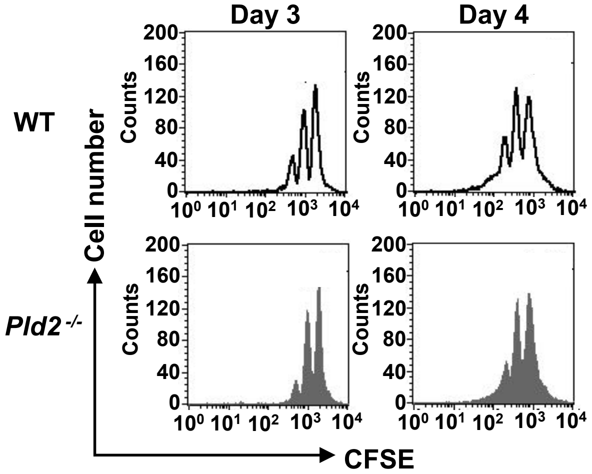

Supplementary Figure S9. **PLD2 is not required for proliferation of CD4^{+} T cells *in vitro*.** Proliferation of splenic CD4^{+} T cells isolated from WT and *Pld2*$^{-/-}$ was assessed as in Figure 6a. Shown are representative of two independent experiments (n=4 for each group).

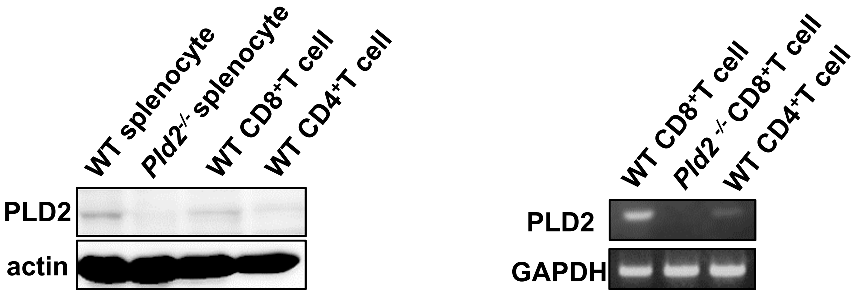

Supplementary Figure S10. Expression level of PLD2 in CD8$^+$ T cells is higher than that in CD4$^+$ T cells. Expression levels of PLD2 protein (left) and mRNA (right) in CD4$^+$ T cells and CD8$^+$ T cells were analyzed by Western blotting and RT-PCR, respectively. Representative of two independent experiments are shown.

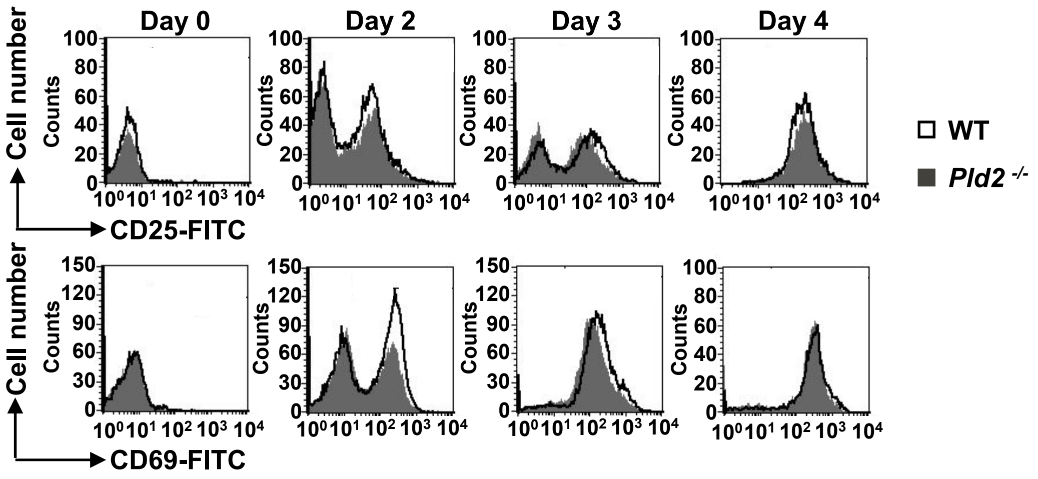

Supplementary Figure S11. PLD2 is not required for activation of splenic CD8^{+} T cells. Splenic CD8^{+} T cells were isolated and stimulated as in Figure 6a. Activation of CD8^{+} T cells was analyzed by the FACS system with the surface markers CD25 (upper panels) and CD69 (lower panels). Shown are the representatives of three independent experiments (n=9 for each group).

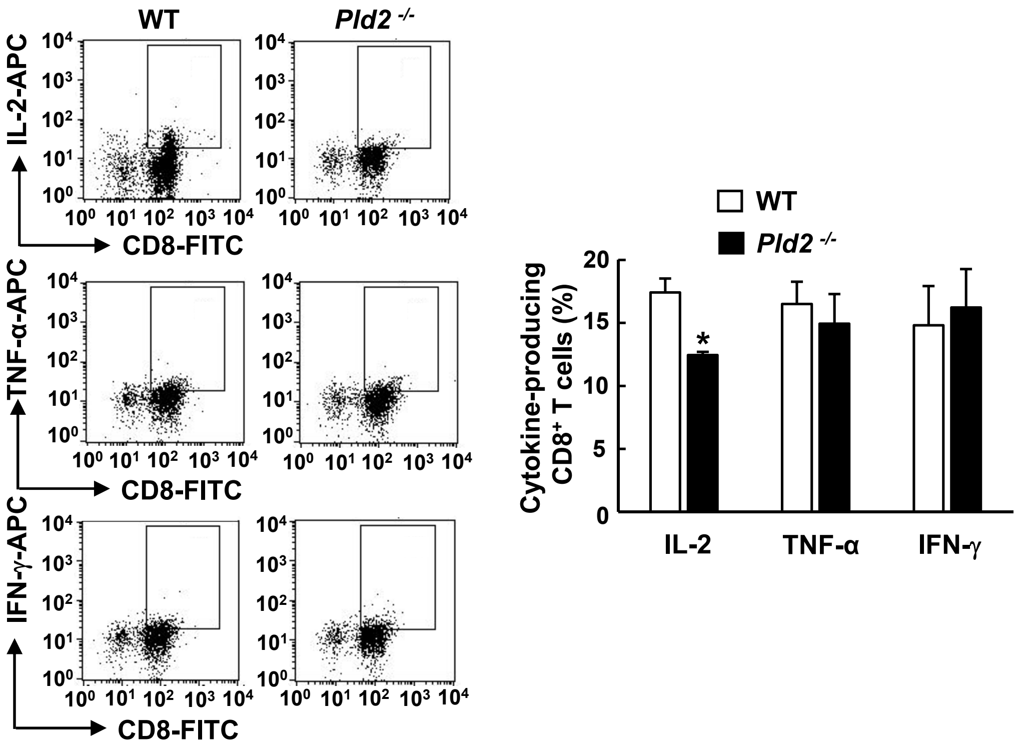

Supplementary Figure S12. CD3/CD28-mediated IL-2 production is impaired in *Pld2*-deleted splenic CD8$^+$ T cells. Splenic CD8$^+$ T cells were stimulated as in Figure 6a for 24 hr. Six hr before the analysis, secretion of cytokines was inhibited by adding Brefeldin A (10 μg/ml), and intracellular IL-2, TNF-α and IFN-γ were labeled with specific antibodies and analyzed by flow cytometry (left). Boxed areas represent CD8$^+$ T cells producing each cytokine. Means ± SEM from three independent experiments are shown in the right graph (n=9 for each group). *p<0.05.

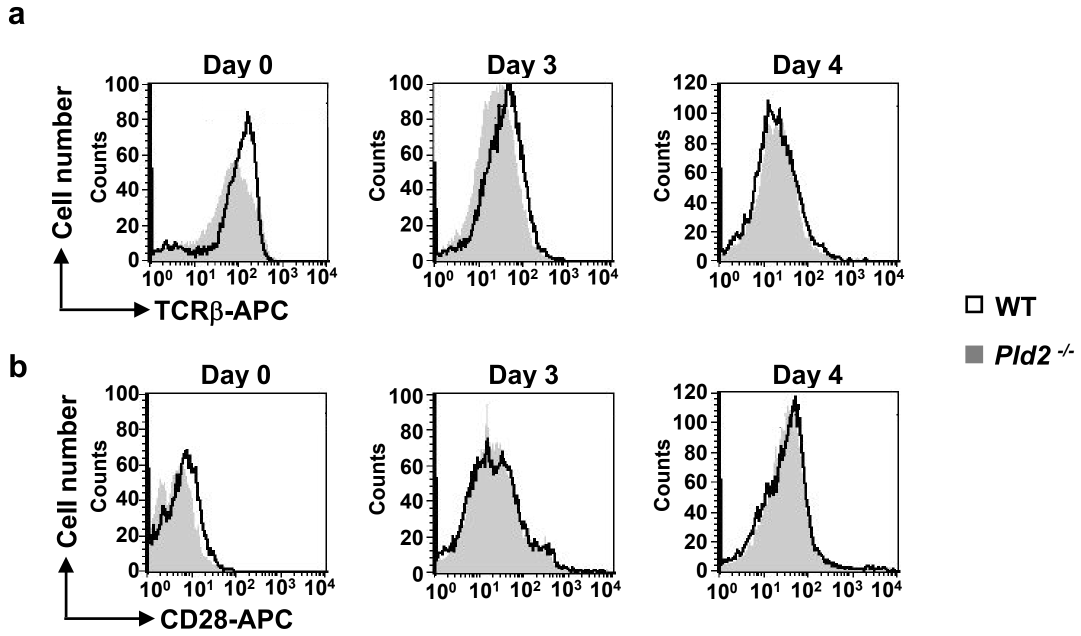

Supplementary Figure S13. Surface levels of TCR and CD28 in WT and *Pld2*$^{-/-}$ splenic CD8^{+} T cells are comparable. WT and *Pld2*$^{-/-}$ splenic CD8^{+} T cells were stimulated as in Figure 6a. Cell surface TCR and CD28 were labeled with antibodies against TCRβ and CD28, respectively, and analyzed by flow cytometry.

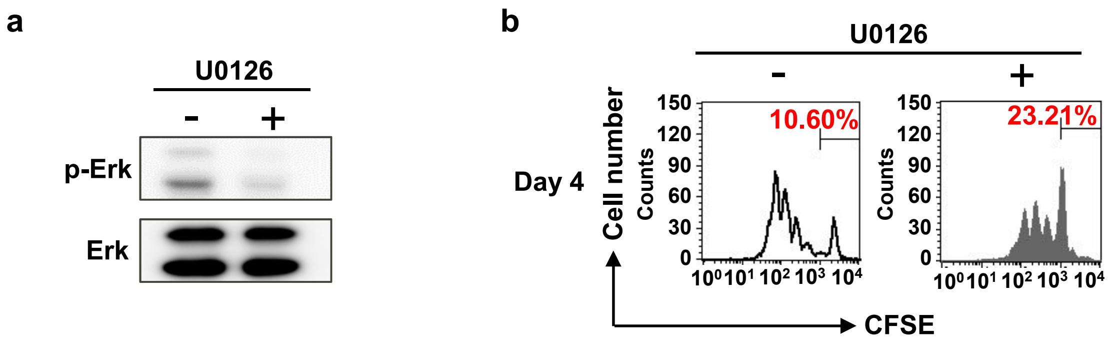

Supplementary Figure S14. Inhibition of Erk phosphorylation suppresses CD3/CD28-stimulated proliferation of splenic CD8$^+$ T cells. (**a**) Splenic T cells were pretreated with or without 5 μM U0126 for 2 hr, and stimulated as in Figure 6a for 30 min. Phosphorylation of Erk was analyzed by Western blotting. (**b**) Splenic T cells were treated with or without U0126 as in (a), and their proliferation was analyzed as in Figure 6a. DMSO was used as a control. The number shown in red in each panel represents the percentage of CFSE-positive cells.

Fig. 7-a

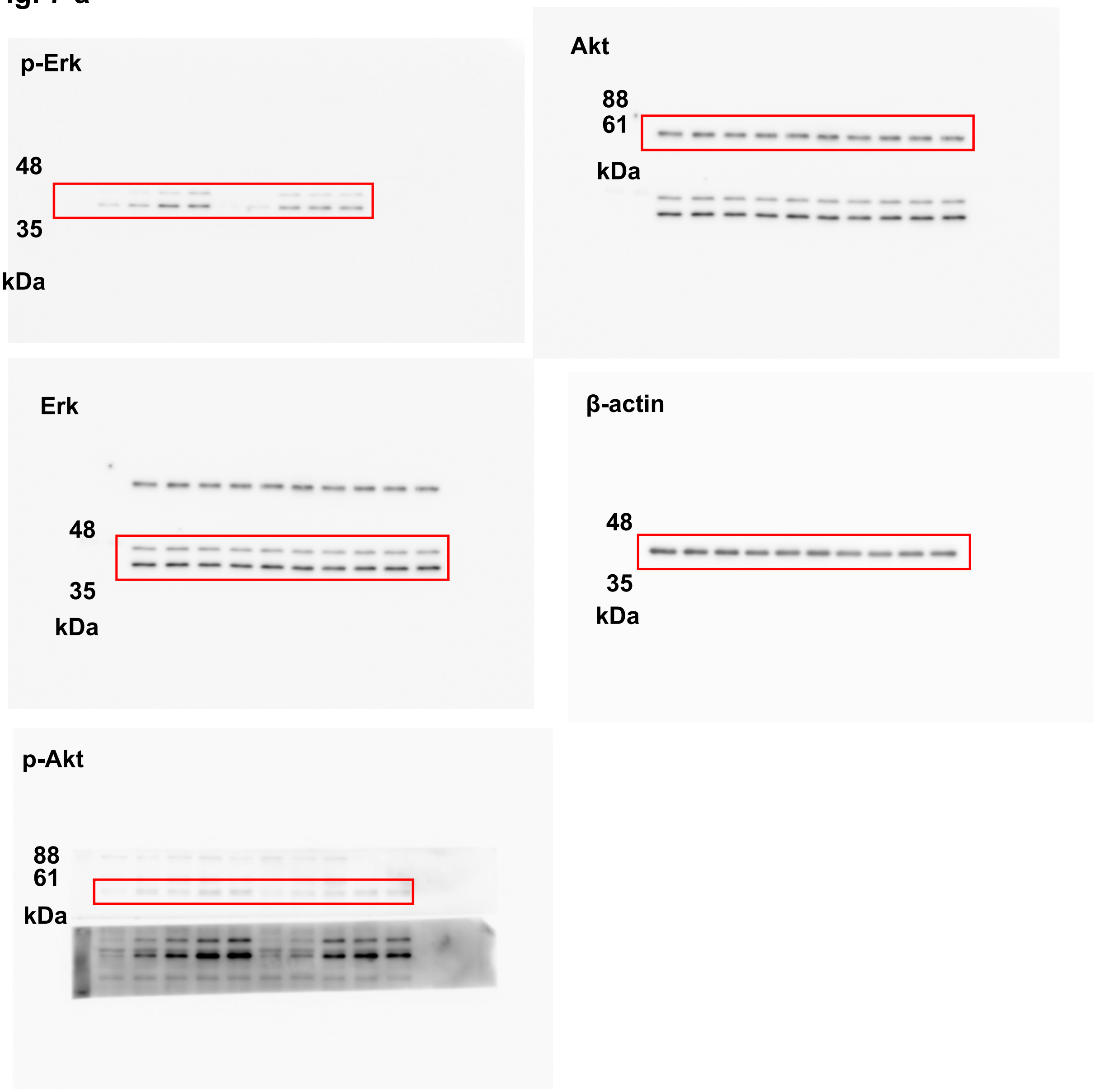

Fig. 7-c

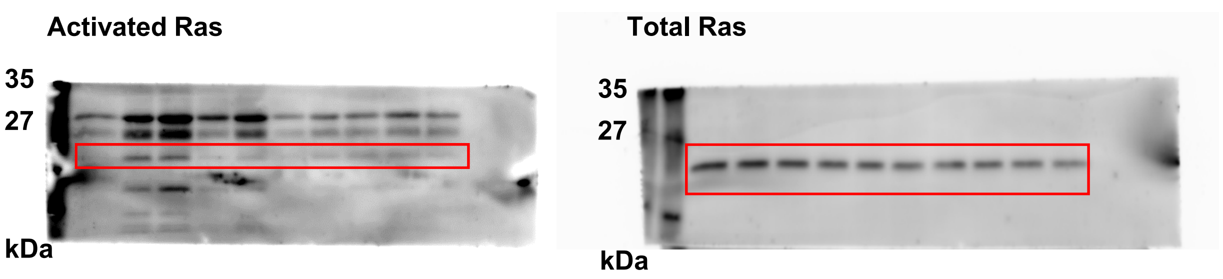

Supplementary Figure S15. Uncropped images of blots and gels.

Supplementary Fig. S2-c

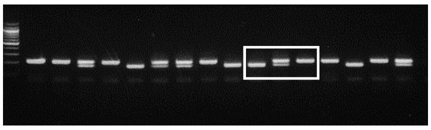

Supplementary Fig. S2-d

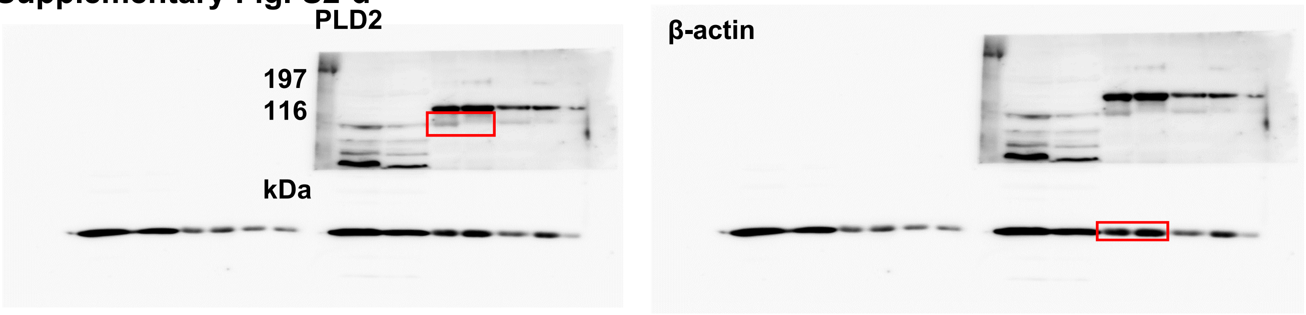

Supplementary Fig. S10

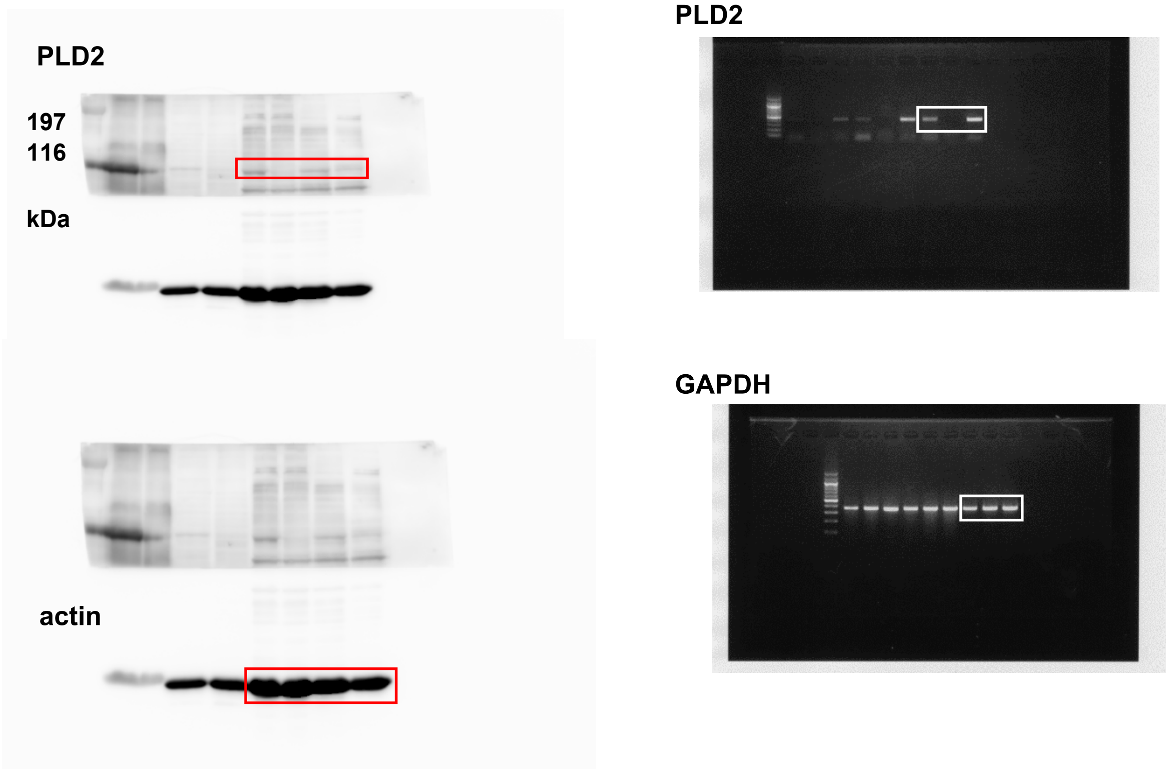

Supplementary Fig. S14-a

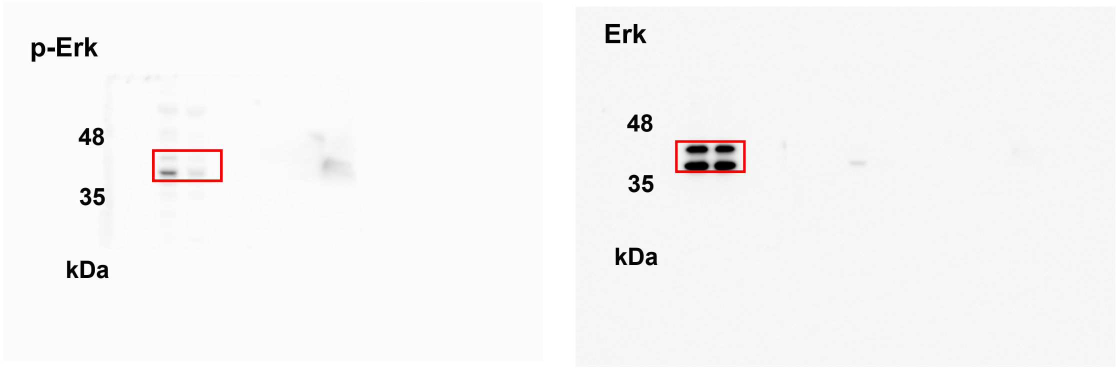

Supplementary Figure S15. Uncropped images of blots and gels.
